# Transdermal Delivery of an mRNA‐Liposome Vaccine via Dissolving Microneedle to Preserve Vaccine Activity and Enhance Immune Activation

**DOI:** 10.1002/advs.202522846

**Published:** 2026-04-09

**Authors:** Jeehye Nam, JiWon Ahn, Jiwoo Shin, Nahong Lee, Youjin Lee, Sung min Cho, Paul Kim, Geonwoo Kang, Sang‐Jun Ha, Hyungil Jung

**Affiliations:** ^1^ Department of Biotechnology Yonsei University Seoul Republic of Korea; ^2^ Department of Biochemistry Yonsei University Seoul Republic of Korea; ^3^ Department of Integrative Biotechnology Yonsei University Inchon Republic of Korea; ^4^ JUVIC Inc. Seoul Republic of Korea

**Keywords:** adaptive immunity, cell‐mediated immunity, dissolving microneedle, innate immunity, mRNA vaccine

## Abstract

Although mRNA vaccines have revolutionized modern vaccinology, significant challenges persist owing to their dependence on ultra‐cold storage, patient discomfort, and logistical complications associated with intramuscular (IM) injections. Dissolving microneedles (DMNs) are a promising alternative by enabling minimally invasive transdermal administration that directly engages skin‐resident immune cells, while their fabrication compromises fragile mRNA. Moreover, previous studies have emphasized antibody‐mediated immunity rather than exploiting their capacity for strong T cell responses. In this study, we developed an mRNA‐liposome vaccine via DMN (ML‐DMN) to preserve the mRNA activity in DMN formulations and boost immune cell activation. Incorporating excipients into the formulation retained the mRNA integrity during fabrication, as demonstrated by in vitro luciferase assays. In vivo administration of ovalbumin (OVA) mRNA via ML‐DMN promoted rapid migration and activation of antigen‐presenting cells (APCs) in draining lymph nodes (dLNs) within 24 h, initiating an innate immune response. This led to robust OVA‐specific T cell activation, proliferation, and secretion of key cytokines, confirming a potent adaptive immune response with lower mRNA doses than IM injections. These results suggest that ML‐DMN systems have significant potential to overcome current mRNA vaccine limitations by improving mRNA integration and immune cell targeting, offering a viable strategy for future vaccine development.

## Graphical abstract

An mRNA‐liposome vaccine via dissolving microneedles (ML‐DMN) enables efficient skin‐targeted delivery while preserving mRNA integrity and allowing sustained antigen expression. Unlike intramuscular (IM) injection, ML‐DMN engages skin‐resident immune cells, activates antigen‐presenting cells (APCs), and induces strong T cell and cytokine responses at reduced doses, highlighting its potential as an innovative, dose‐sparing vaccination strategy.

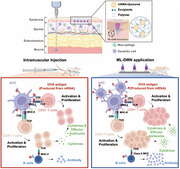

## 1 Introduction

The success of mRNA vaccines against SARS‐CoV‐2 has revolutionized the field of vaccinology, opening a new era for disease prevention and treatment [1]. mRNA vaccines have several advantages, such as no risk of genome integration and the capability of targeting multiple antigens [2, 3]. Furthermore, as the engineering and synthesis processes of mRNA vaccines are simple and quick, they can be mass‐produced in a short period, which is highly advantageous for responding to pandemic situations [2, 3, 4, 5, 6, 7, 8, 9]. Additionally, mRNA vaccination naturally produces endogenous antigens that can induce a stronger CD8^+^ T cell response than protein‐based vaccination, thus demonstrating superior vaccine efficacy [1, 10]. Although these advantages have fueled rapid industrial growth, major hindrances remain in optimizing cellular uptake and preserving mRNA from degradation [3, 11].

Therefore, drug carriers such as liposomes, lipid nanoparticles (LNP), and polymer‐based nanoparticles were introduced to preserve mRNA stability by effectively delivering mRNA into cells and protecting it from ribonucleases (RNase) [12, 13]. Among these, mRNA‐LNP vaccines, which are widely used as drug carriers for various formats and administrations, offer prompt immunogenicity, with several candidates proving effective in clinical trials [14, 15]. This has led to the widespread adoption of LNPs in mRNA vaccine development, including COVID‐19 vaccines, thereby mitigating the global impact of the COVID‐19 pandemic. Recent advances in LNP engineering have further improved encapsulation efficiency, endosomal escape, and in vivo stability, solidifying LNPs as the current gold standard for mRNA delivery [16, 17, 18]. Still, their reliance on ultra‐cold storage complicates vaccine distribution in resource‐limited countries, leading to disparities in vaccine availability [3, 19, 20]. mRNA‐LNP vaccination also uses traditional invasive intramuscular (IM) injections, which bypass the dermis, an area abundant in immune cells, thus limiting the immune response efficiency [21]. Additionally, the thawing and mixing procedures increase the risk of errors during administration by healthcare professionals.

Dissolving microneedles (DMNs) are promising alternatives to injections by addressing these obstacles. Enhanced thermostability through solid antigen encapsulation prevents antigen degradation, enabling room‐temperature distribution in low‐resource environments [22, 23, 24]. mRNA activity is further conserved by combining polymers and excipients such as trehalose and sucrose within the DMN matrix [25, 26, 27, 28]. DMNs also offer a painless and minimally invasive method for delivering vaccines by directly targeting skin‐resident immune cells, achieving potent immune responses with reduced mRNA doses [29, 30, 31, 32, 33]. In addition, self‐administration of DMN eliminates handling complexity and biohazardous waste, thereby enhancing its practicality and accessibility.

Although mRNA‐loaded DMNs offer promising advantages for mRNA delivery, the inherent instability of mRNA during DMN fabrication poses a significant barrier [34, 35]. For example, the inevitable drying and solidification process for DMN formation exposes intrinsically fragile mRNA to harsh conditions, accelerating degradation, and resulting in the restricted application of DMNs in mRNA vaccine innovation. Owing to mRNA instability, Yu et al. developed a cryomicroneedle system using cryogenic techniques to stabilize mRNA against degradation with polymer‐based nanoparticles, showing IFN‐γ‐secreting T cells and antibody titers [36]. As this cryomicroneedle required −80°C storage, its practical application was limited by logistical challenges, particularly in resource‐constrained areas. Straeten et al. addressed these by utilizing LNP as carriers for mRNA vaccines via DMNs to achieve room‐temperature stability and induce humoral immune responses [37]. MN‐based mRNA–LNP platforms have further advanced transdermal vaccination by improving formulation robustness, storage stability, and in vivo immunogenicity [37, 38, 39]. Although these studies demonstrated technical feasibility and protective immune responses, systematic and condition‐matched quantitative comparisons of immune performance across delivery routes remain limited. Moreover, these prior studies primarily focused on antibody‐mediated outcomes, with comparatively less emphasis on antigen‐specific T cell responses enabled by direct targeting of skin‐resident immune cells, which are essential for triggering cell‐mediated immunity across both innate and adaptive compartments. This is particularly significant as T cell‐driven responses are essential for adaptive immunity, providing defense against intracellular pathogens, and supporting long‐term immune memory [40, 41]. Recognizing the importance of both humoral and cell‐mediated immunity in vaccine efficacy, the European Medicines Agency (EMA) guidelines emphasize the necessity of assessing both immune pathways in novel vaccine research [42]. Therefore, the integration of comprehensive evaluations of these immune responses is essential for advancing the development of effective and broadly protective vaccination strategies.

In this study, we developed an mRNA‐liposome vaccine via DMN (ML‐DMN) to improve the incorporation of mRNA into DMNs and activate antigen‐presenting cells (APCs) and T cells, thereby eliciting both antibody‐ and cell‐mediated immune responses. The ML‐DMN was designed with a multilayer structure, in which the core layer encapsulated the mRNA‐liposome, the shell layer ensured protection of the core and skin penetration, and the base layer provided structural support, all working together to facilitate effective delivery [43, 44]. In vitro and in vivo luciferase assays validated that the combination of trehalose and sucrose in the optimized core formulation preserved mRNA potency during fabrication. The administration of ovalbumin (OVA) mRNA via ML‐DMN significantly activated macrophages and conventional dendritic cells (cDCs) in the draining lymph nodes (dLNs) within 24 h. This enhanced innate immune response triggered the activation of OVA‐specific CD8^+^ and CD4^+^ T cells, leading to humoral and cellular immunity compared to IM injections. Therefore, overcoming the limitations of conventional mRNA vaccines, ML‐DMN represents a novel approach that utilizes mRNA‐liposomes to achieve stability at 4°C, simplifying storage and targeting immune cell‐rich skin environments. This strategy drove strong T cell immune responses, ultimately demonstrating a clear dose‐sparing effect. Moreover, ML‐DMN has a significant potential to advance mRNA safety and effectiveness, expanding the possibilities for more reliable and impactful vaccination in the future.

## 2 Results and Discussion

### 2.1 ML‐DMN for Innate and Adaptive Immune Responses

**FIGURE 1:**
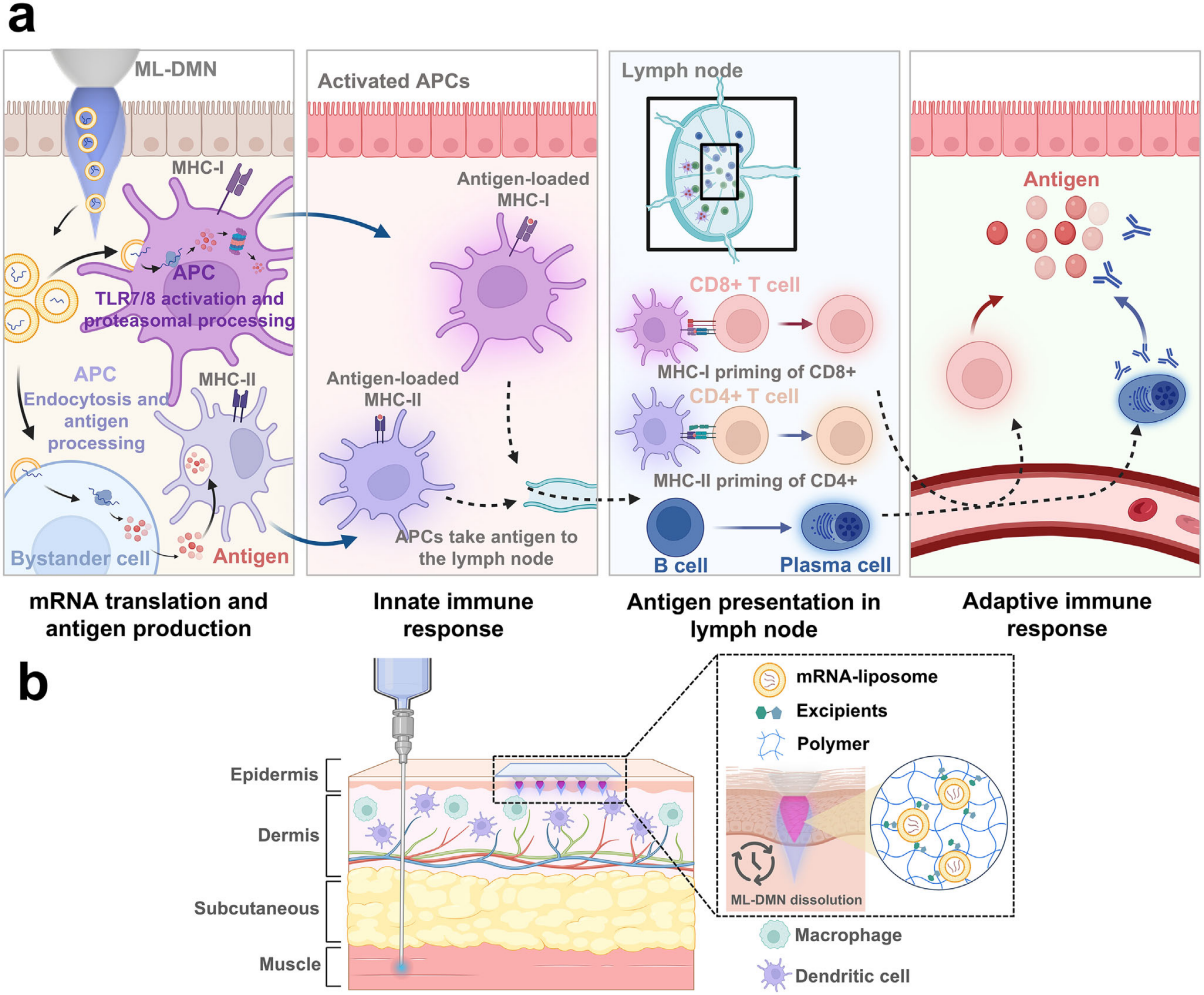
Comparative analysis of immune responses induced by mRNA‐liposome via ML‐DMN and IM Injection. (a) Schematic diagram of ML‐DMN application for innate and adaptive immunity. (b) Conceptual illustration comparing how IM injection and ML‐DMN application deliver mRNA‐liposome through distinct anatomical routes.

The innate and adaptive immune responses of ML‐DMN are shown in Figure 1a. Following the direct penetration of ML‐DMN into the dermis through the epidermis of the skin, the dissolution of ML‐DMN releases mRNA‐liposomes, resulting in the delivery of mRNA to the cytosol of various cells localized in the dermis and its translation into antigen proteins (Figure 1a). APCs, such as DCs and macrophages within the dermis, are activated upon uptake of mRNA and present newly produced and processed antigens to major histocompatibility complex (MHC) class I molecules. In addition, other cells within the dermis can produce antigen proteins, which are also endocytosed, processed, and presented to MHC class II molecules in APCs. Subsequently, APCs drain into the LNs, where they prime antigen‐specific CD8^+^ and/or CD4^+^ T cells and stimulate their proliferation and differentiation. Some activated CD4^+^ T cells help B cells in the LNs, leading to their differentiation into plasma cells. These adaptive immune responses are characterized by the production of cytokines by proliferating T cells and the production of antibodies by plasma cells.

Based on this initial immune response, a comparative analysis between the IM injection and ML‐DMN application of the mRNA vaccine revealed notable differences in immune activation (Figure 1b). IM injection involves deeper tissue penetration, where fewer APCs are present, resulting in less efficient antigen uptake and a comparatively weaker immune response [45]. In contrast, the ML‐DMN system enabled more effective engagement of abundant skin‐resident APCs within the dermal microenvironment, thereby maximizing the immunogenic potential of mRNA. Furthermore, the ML‐DMN system facilitated prolonged antigen expression through the polymer matrix, which gradually dissolved and released mRNA, enabling sustained activation of APCs compared with IM injection. This localized delivery enhanced downstream T cell proliferation, cytokine secretion, and antigen‐specific antibody production, demonstrating the superior capacity of ML‐DMN to stimulate both cell‐mediated and humoral immune responses. This system offered improved efficacy over traditional injection methods and may serve as a candidate for future vaccine development.

### 2.2 Fabrication and Structural In Vitro Insertion Analysis of ML‐DMN

**FIGURE 2:**
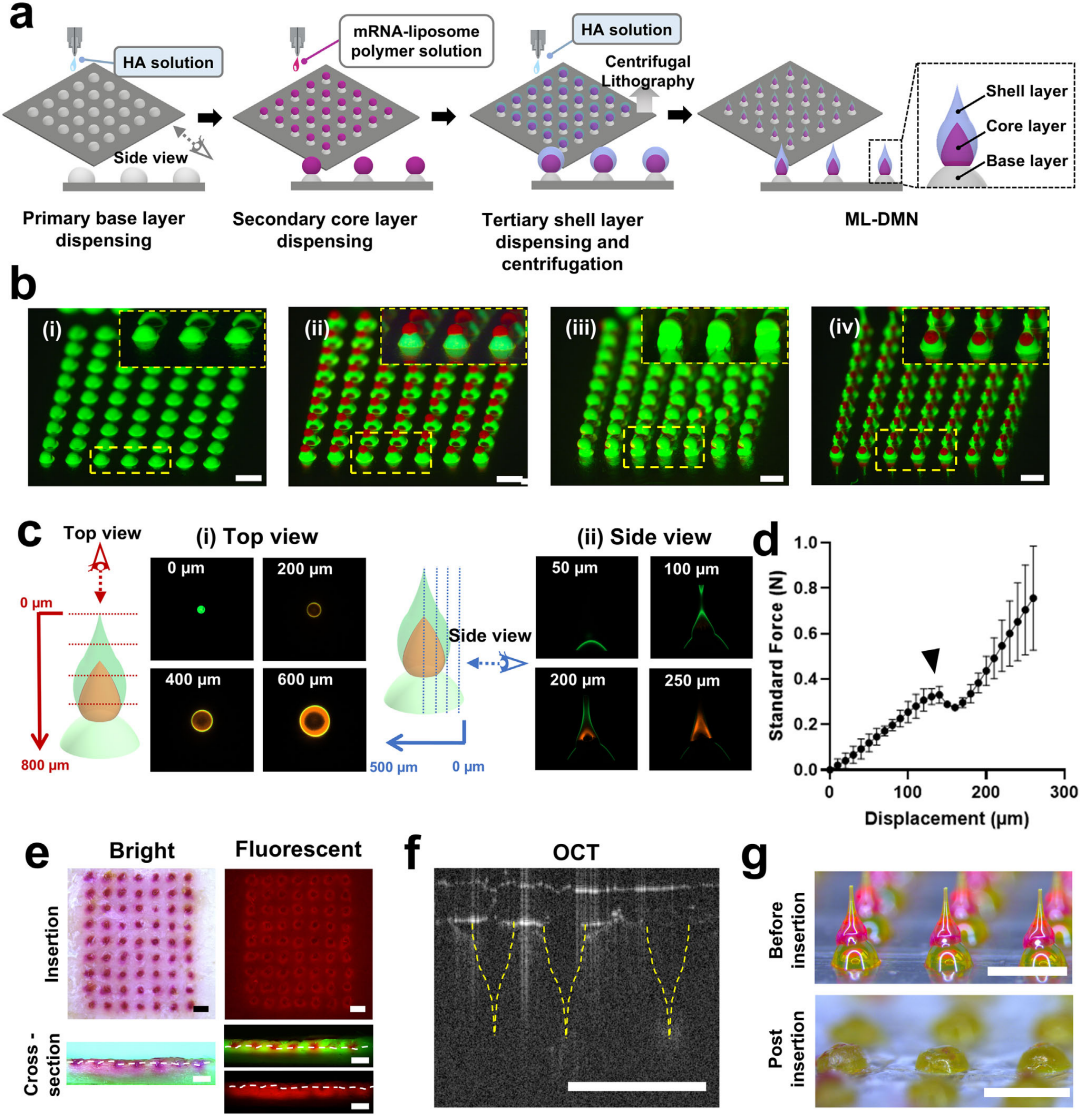
Schematic representation of ML‐DMN fabrication, structural evaluation, and skin penetration analysis. (a) Fabrication process of ML‐DMN using sequential layer dispensing and centrifugal lithography. (b) Fluorescent imaging of the ML‐DMN fabrication stages: (i) base layer (calcein), (ii) core layer (rhodamine B), (iii) shell layer (calcein), and (iv) fully fabricated ML‐DMN after lithography (scale bar, 1 mm). (c) Confocal microscopy analysis of fluorescent ML‐DMN structure. (i) Top view cross‐sectional imaging at 200 µm intervals and (ii) side view cross‐sectional imaging at 50, 100, 200, and 250 µm showing the distribution of the base, core, and shell layers. (d) Fracture force analysis of ML‐DMN mechanical strength. Force–displacement curves are presented as mean ± SEM (n = 3). (e) In vitro skin penetration test using fluorescent ML‐DMNs on porcine skin. Top and cross‐sectional view of bright field and fluorescent images after 15 min of application (scale bar, 1 mm). (f) Optical coherence tomography (OCT) imaging of ML‐DMN (yellow dotted lines) insertion into porcine skin (scale bar, 1 mm). (g) Residue analysis before and post fluorescent ML‐DMN application (scale bar, 1 mm).

The ML‐DMN was designed to ensure stable and precise delivery of mRNA‐liposomes to the skin with three distinct layers, including a base, core, and shell layer, as illustrated in Figure 2a, which shows the fabrication process involving the sequential dispensing of these layers. The first dispensed base layer, composed solely of hyaluronic acid (HA) solution, was developed to ensure effective mRNA delivery. This base layer was designed to be approximately 300 µm in height, providing the necessary structural support for the complete insertion of the mRNA‐liposome‐loaded core into the skin [43, 44]. Next, the core layer containing the mRNA‐liposomes was carefully dispensed onto the base layer using an optimized formulation developed through the systematic screening of polymers and excipients. This formulation ensured the activity of the mRNA‐liposomes during fabrication and preserved their bioactivity for effective post‐delivery expression. The final shell layer, composed of an HA solution without mRNA‐liposomes, was then dispensed to cover the core layer, protect it from external stress, and preserve mRNA‐liposome activity during fabrication [44]. The shell layer underwent centrifugal lithography, in which the centrifugal force stretched the viscous layer, forming the final shape of the ML‐DMNs [46].

To visualize the fabrication stages, green calcein was used for the base and shell, while red rhodamine B marked the core layer. Fluorescent images (Figure 2b) revealed that the green‐base layer on the custom‐made patch substrate (Figure 2b‐i), the red‐core layer was located on the base layer (Figure 2b‐ii), and the green‐shell layer covered both (Figure 2b‐iii). Fluorescent ML‐DMNs were successfully formed after centrifugal lithography (Figure 2b‐iv). Confocal imaging was used to observe each layer and comprehensively verify the structure of the ML‐DMNs (Figure 2c). The top view of the z‐stack images was obtained by setting the tip of the ML‐DMN as the reference point and capturing images at 200 µm intervals. Initially, green fluorescence, indicating the shell layer, was visible in the top view (Figure 2c‐i). At 200, 400, and 600 µm depths, the core structure stained with rhodamine B and the surrounding shell layer were simultaneously observed. Diffusion of the orange‐core layer into the green‐shell layer was observed at the interface. Sequential imaging demonstrated that the base, core, and shell layers remained distinct and structurally intact, supporting their specific functional roles in ML‐DMN formation. Using the same method, the side view provided further insight into the layered structure of the ML‐DMN (Figure 2c‐ii). Cross‐sectional images taken at 50, 100, 200, and 250 µm revealed a clear separation of the base, core, and shell layers. Green fluorescence from the base was detected at the initial depths, followed by that from the core and shell layers as the depth increased. This view reinforces the distinct and organized architecture of ML‐DMN and highlights the functional independence of each layer.

Fracture force and in vitro skin penetration tests were performed to confirm the effective delivery of mRNA‐liposomes by the ML‐DMNs. The fracture force analyzer measured the axial force applied by a vertically downward probe, finding that a single ML‐DMN could withstand 0.33 ± 0.02 N of force, sufficient for skin penetration with a similar shape (Figure 2d) [47]. For the in vitro skin penetration test, fluorescent ML‐DMNs were applied to the porcine skin using a custom‐made applicator. After 15 min of application, 7 × 9 ML‐DMN array perforations were visible, with bright field and fluorescent images verifying skin penetration (Figure 2e). Cross‐sectional imaging revealed the widespread diffusion of red rhodamine B surrounded by green calcein, demonstrating that the green base layer provided additional height to the red core layer, allowing complete detachment from the patch surface and successful insertion into the skin. Additionally, optical coherence tomography (OCT) imaging further confirmed the effective penetration of ML‐DMNs (yellow dotted lines) into the pig skin (Figure 2f). Bright‐field images captured before and 15 min after patch application showed that the calcein‐loaded base residue remained, with no detectable rhodamine B‐loaded core (Figure 2g). The combined assessment of perforations, OCT imaging, and residue analysis collectively verified the successful delivery of the core layer to pig skin.

### 2.3 In Vitro Analysis of mRNA‐Liposome Activity

As ML‐DMN was fabricated from viscous mixtures of mRNA‐liposomes and polymers, the mixing process involved fluctuations in temperature, pH, and chemical structure, often leading to a loss of mRNA potency and the structural integrity of liposomes. Furthermore, the unavoidable drying process, which provided sufficient mechanical strength for skin insertion, often resulted in dehydration‐related stresses and phase transitions, which further reduced the maintenance of mRNA‐liposome functionality.

**FIGURE 3:**
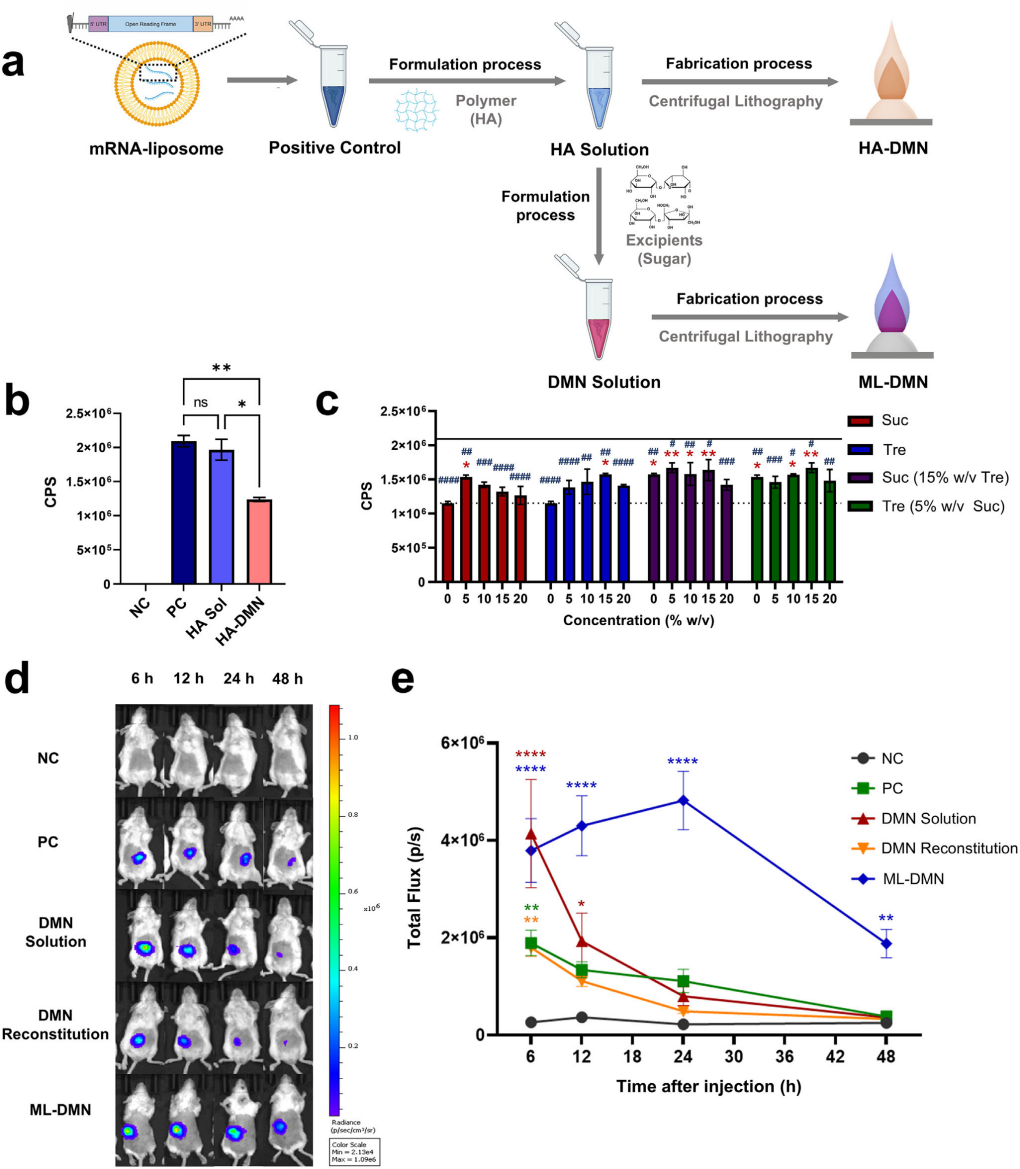
Luciferase expression and activity evaluation, both in vitro and in vivo, using IVIS imaging to demonstrate mRNA delivery efficiency and expression kinetics. (a) Schematic of ML‐DMN formulation and fabrication process. (b) Luciferase activity of mRNA‐liposomes measured in the J774a.1 cell line: NC, PC, HA solution, and HA‐DMN (n = 3). (c) Optimization of trehalose and sucrose concentrations for maximum mRNA activity retention post‐drying and fabrication (n = 3). (d) IVIS imaging of luciferase mRNA expression in mice at various time points (6, 12, 24, and 48 h) following mRNA‐liposomes administration via ML‐DMNs and ID injection. (e) Total flux quantification of luciferase expression over 48 h, showing luminescence kinetics for different delivery methods, including NC, PC, DMN solution, DMN reconstitution, and ML‐DMN (n = 3). Kinetics and column graphs show mean ± SEM. Statistical significance was analyzed by one‐way ANOVA followed by Tukey's multiple comparison test for (b) and Dunnett's multiple comparison test for (c,e). In (c), HA‐DMN and PC were used as reference groups for * and # annotations, respectively. In (e), NC served as the reference group. ns, not statistically significant; * and #, *p* < 0.05, ^**^ and ##, *p* < 0.01, ^***^ and ###, *p* < 0.001, ^****^ and ####, *p* < 0.0001.

We optimized the composition of the core layer in ML‐DMNs to preserve the activity of mRNA‐liposome throughout both the formulation and fabrication processes by screening excipients [20, 48]. Figure 3a illustrates the detailed formulation and preparation of the mRNA‐liposomes incorporated within the ML‐DMNs. The process began with the synthesis of mRNA‐liposomes, which served as a positive control (PC). The mRNA‐liposomes were mixed with HA, and solutions that underwent only this formulation process were labeled as the HA solution, whereas those subjected to drying and fabrication of DMN without excipients were labeled as HA‐DMN. Additionally, the formulation process combined HA solutions with stabilizing excipients, such as trehalose and sucrose, to prepare a DMN solution, which was dispensed and fabricated using the centrifugal lithography method to fabricate the ML‐DMN.

First, the functional activity of the mRNA was evaluated using luciferase mRNA‐liposomes in the J774a.1 cell line derived from mouse macrophages using luciferase assays (Figure 3b). The negative control (NC), which consisted of PBS, showed no detectable luciferase expression, confirming the specificity of the assay. The PC and HA solution groups exhibited similar expression levels, indicating a minimal loss of mRNA activity during the formulation process. Conversely, luciferase expression in the HA‐DMN samples dropped significantly to 55.1% ± 2.6% of that in the PC group, highlighting the challenge of the drying process in reducing mRNA‐liposome potency. To minimize mRNA activity loss in the HA‐DMN group caused by the inevitable process, saccharide‐based stabilizers, such as sucrose and trehalose, were optimized based on the water replacement theory, which states that saccharides bind to the polar head groups of lipids to replace water and stabilize the structure during drying and storage (Figure 3c) [24, 25, 26, 27]. We applied ANOVA and used post‐hoc Dunnett's multiple comparison test to identify significant differences between the HA‐DMN group and the various excipient formulation groups. Sucrose at 5% (w/v) and trehalose at 15% (w/v) showed the highest luminescence values, both showing statistically significant improvements (^*^ *p* < 0.05) compared to the HA‐DMN group (dashed line). Further combination of screening revealed a higher mRNA‐liposome activity with 15% (w/v) trehalose and 5% (w/v) sucrose (Figure 3c). Additionally, statistical comparisons against the PC group were performed using Dunnett's multiple comparisons test, showing that all excipient‐formulated conditions, including the optimized formulation, were statistically distinguishable from the PC reference (*p*< 0.05). In this context, the observed activity level of 78.3% ± 4.6% of PC reflects substantial preservation of mRNA functionality relative to HA‐DMN within the solid‐state formulation.

After optimization of the formulation components, the structural integrity of the mRNA‐liposomes in the PC group was initially assessed using dynamic light scattering (DLS) without undergoing the formulation and fabrication processes (Figure S1a). The DLS analysis confirmed that the liposomes maintained a consistent size with an average diameter of 223.7 ± 2.2 nm and a polydispersity index (PDI) of 0.177 ± 0.014. The PDI value reflected a narrow size distribution, ensuring minimal aggregation and consistent incorporation of mRNA‐liposomes. Subsequently, transmission electron microscopy (TEM) analysis revealed that the liposomes remained intact and well defined in the PC sample, within the DMN matrix, and in the final ML‐DMN, even after the formulation and fabrication steps. (Figure S1b). These results demonstrated the protective effects of the excipients, effectively maintaining the potency and structural integrity of mRNA‐liposomes during the drying process.

### 2.4 In Vivo Evaluation of mRNA Transfection Efficiency with ML‐DMN via IVIS Analysis

After refining the formulation composition through in vitro studies, the in vivo efficiency of mRNA expression via a transdermal system was evaluated using IVIS analysis following the administration of luciferase mRNA‐liposome (Figure 3d,e). This IVIS experiment was designed to evaluate relative in vivo expression kinetics and temporal persistence of mRNA delivery systems, rather than to provide a direct quantitative comparison of absolute signal intensity across different anatomical conditions. To enable comparable evaluation of mRNA expression across delivery methods, ID injection was performed to approximate the release depth of ML‐DMN in the dermal layer. IVIS signal intensity was recognized to be influenced by anatomical and physiological variables, including tissue thickness, optical attenuation, and local subcutaneous blood flow. Accordingly, signal magnitude was not interpreted as a standalone indicator of delivery efficiency. The experimental groups included NC treated with PBS; PC containing firefly luciferase mRNA‐liposomes; a DMN solution incorporating mRNA‐liposomes along with HA, trehalose, and sucrose; DMN reconstitution made by dissolving ML‐DMN in PBS for ID injection; and ML‐DMN applied directly through the insertion of mouse dorsal skin. The DMN reconstitution group served as a reference to verify whether incomplete insertion could lead to variability in the results, ensuring a reliable assessment of DMN‐specific performance.

Upon examining the results, the PC group exhibited lower luminescence compared to the DMN solution group, emphasizing the role of polymers and excipients in stabilizing mRNA and enhancing its expression in the DMN solution formulation (Figure 3e). Additionally, the DMN reconstitution group showed diminished luminescence in contrast to the DMN solution group, likely due to the fabrication process compromising the preservation of mRNA‐liposomes.

Conversely, although the other groups showed a dramatic decrease in luminescence, the ML‐DMN group maintained a significantly higher signal, persisting for up to 48 h (Figure 3e). This prolonged luminescence, observed relative to all injection groups including the DMN reconstitution group, was interpreted as evidence of sustained expression kinetics rather than absolute signal superiority, as all groups were analyzed under identical imaging parameters, while potential variability arising from differences in anatomical administration sites and associated physiological factors was taken into consideration. This observation suggested that differences in the delivery route and local release kinetics contributed to the prolonged mRNA expression observed in the ML‐DMN group. Injection groups resulted in localized deposition with rapid diffusion driven by steep concentration gradients, whereas ML‐DMN, upon application, gradually dissolved within the dermis through its matrix, enabling sustained release of the encapsulated mRNA over time, which maintained functional activity. This gradual release ensured continuous antigen production, allowing prolonged exposure of the immune system to the antigen. Collectively, these results indicated that ML‐DMN enabled sustained in vivo mRNA expression, thereby promoting extended antigen availability over time and potentially enhancing both the magnitude and duration of the immune response.

### 2.5 In Vivo Evaluation of Innate Immune Cell Response

Given the proven efficiency of ML‐DMN in mRNA delivery and its ability to sustain activity through luciferase assays, we aimed to assess its potential to amplify vaccine‐induced immune responses. Supported by the evidence of DMN patches as delivery platforms for protein‐and nucleic acid‐based vaccines targeting the dermal innate immune system, ML‐DMN vaccines were expected to enhance these early innate immune responses, which are critical for initiating antigen recognition, activating APCs, and providing cytokine signaling to bridge adaptive immunity [49, 50, 51, 52, 53]. To validate this, we investigated the innate immune cells present in mouse dLNs near the vaccination sites at early time points post‐vaccination using flow cytometry gating strategies, as detailed in Figure S2 [54, 55].

**FIGURE 4:**
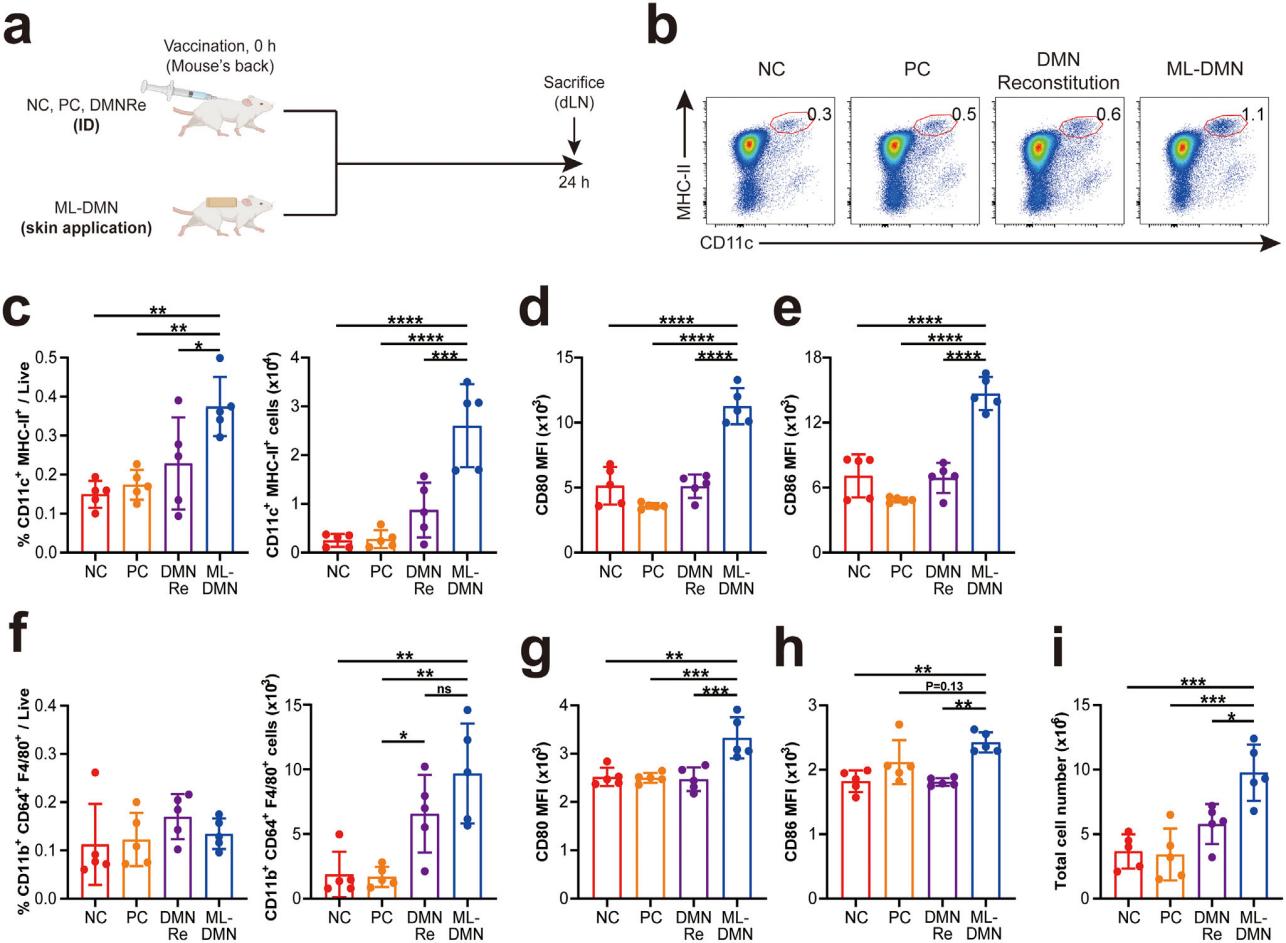
In vivo experimental analysis of innate immune responses mediated by macrophages and cDCs in the dLN. (a) In vivo experimental scheme for analysis of vaccine‐induced innate immune responses at early time points. Naïve mice (C57BL/6) were immunized with various forms of mRNA vaccines loaded with 1.5 µg OVA mRNA (n = 5 per group). (b) Representative flow plots depicting the frequencies of cDCs in dLNs. The flow plots are gated on Thy1.2^−^ CD19^−^ NK1.1^−^ Siglec‐H^−^ cells. (c) Bar graphs showing the frequencies and absolute numbers of cDCs in dLNs. (d,e) MFI analyses of (d) CD80 and (e) CD86 expression level of cDCs in dLNs. (f) Bar graphs showing the frequencies and absolute numbers of macrophages in dLNs. (g,h) MFI analyses showing (g) CD80 and (h) CD86 expression level of macrophages in dLNs. (i) Total cell number present in dLNs. The data are representative of two independent experiments. Column graphs show mean ± SD. Statistical significance was analyzed by one‐way ANOVA with Tukey's multiple comparisons test. ns, not statistically significant; ^*^ *p* < 0.05; ^**^ *p* < 0.01; ^***^ *p* < 0.001; ^****^ *p* < 0.0001.

To evaluate immune responses in a mouse model, we utilized the model antigen OVA and developed various OVA mRNA vaccine formulations (loaded with 1.5 µg mRNA) for administration in naïve mice. For innate immune response analysis, four groups of mice (NC, PC, DMN reconstitution, and ML‐DMN) were used, with all vaccines applied to the backs of the mice. ML‐DMNs were delivered via patch attachment, whereas ID injections were used for the other groups (Figure 4a). To uniformly target the inguinal LNs, which predominantly receive drainage from the back owing to their anatomical proximity, all vaccines, except ML‐DMN, were administered by ID injection. At 24 h after vaccination, the mice were sacrificed, and innate immune cell populations in the inguinal LNs were analyzed by flow cytometry.

The ML‐DMN group exhibited the highest frequency and number of cDCs among all the experimental groups (Figure 4b,c). In addition, cDCs in the ML‐DMN group showed a marked increase in the expression of co‐stimulatory molecules (CD80 and CD86), indicating enhanced activation (Figure 4d,e). Although there were no significant differences in the frequencies of macrophages between the groups, cell numbers were higher in the ML‐DMN group than in the NC and PC groups (Figure 4f). Likewise, CD80/CD86 expression levels in macrophages were significantly higher in the ML‐DMN group, suggesting increased activation of this population (Figure 4g,h). ML‐DMN also increased the total cell number in dLNs, showing a greater accumulation of immune cells in local lymphoid organs near the vaccination sites (Figure 4i).

These results demonstrated the ability of ML‐DMN to enhance the activation and quantity of innate immune cells, as reflected by higher cDC and macrophage frequencies and elevated expression of CD80/CD86, which are critical for antigen presentation and T cell activation. Furthermore, the increase in total immune cell numbers within the dLNs highlighted enhanced recruitment and activation, promoting a supportive immunological microenvironment. Vaccination with ML‐DMN fostered a robust innate immune foundation, likely driving subsequent adaptive immune responses, and validating its potential as an advanced vaccine platform.

### 2.6 In Vivo Assessment of Adaptive Immune Responses Through Antigen‐Specific T Cell Activity in Peripheral Blood

Based on the observed innate immune activation, we evaluated the ability of the ML‐DMN vaccines to generate effective adaptive immune responses. Four experimental groups (NC, PC, DMN reconstitution, and ML‐DMN) received 1.5 µg OVA mRNA vaccines, with all groups except ML‐DMN administered via IM injection to match the standard vaccination method widely used in clinical practice, facilitating a direct comparison with patch‐based delivery.

**FIGURE 5:**
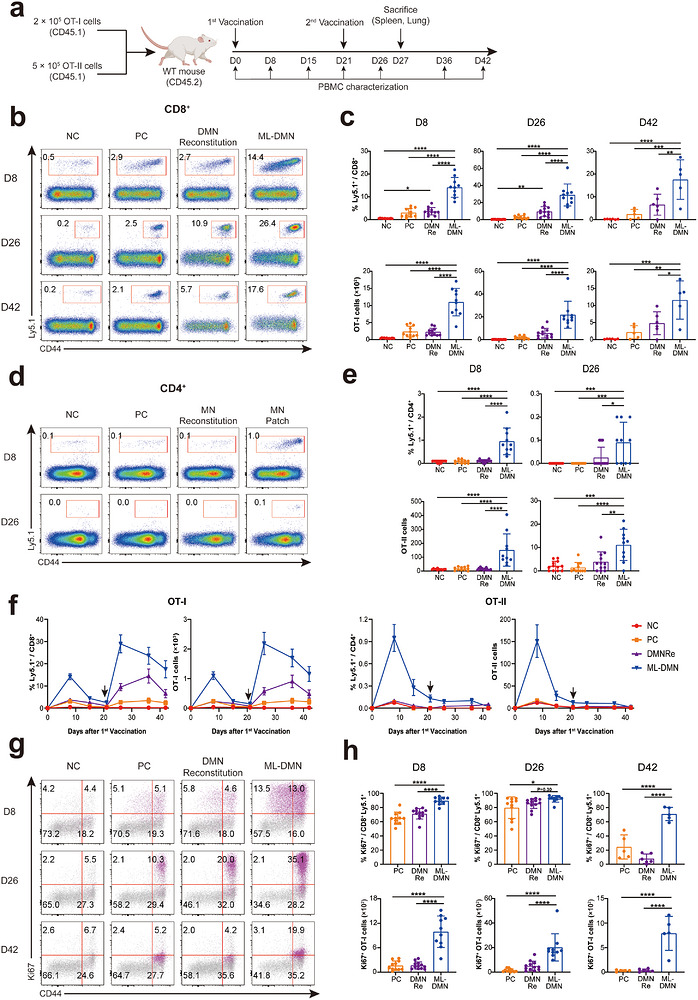
Experimental scheme and PBMC analysis at 1.5 µg OVA mRNA vaccination. (a) In vivo experimental scheme for assessing mRNA vaccine‐induced immune responses. Naïve CD45.2^+^ mice (C57BL/6) were co‐transferred with CD45.1^+^ OVA‐specific OT‐I (CD8^+^) and OT‐II (CD4^+^) cells and then immunized with various forms of mRNA vaccines loaded with 1.5 µg OVA mRNA (n = 10–12 per group). Antigen‐specific T cells in PBMC and organs (spleen and lung) were analyzed by flow cytometry at the indicated time points. (b) Concatenated flow plots showing the frequencies of Ly5.1^+^ OT‐I cells (gated on CD8^+^ T cells) in PBMC on days 8, 26, and 42 after 1st vaccination. (c) Graphs illustrating the frequencies (top) and cell numbers (bottom) of Ly5.1^+^ OT‐I cells per 1 × 10^5^ PBMCs at each time point. (d) Concatenated flow plots depicting the frequencies of Ly5.1^+^ OT‐II cells (gated on CD4^+^ T cells) in PBMC on days 8 and 26 after 1^st^ vaccination. (e) Graphs showing the frequencies (top) and cell numbers (bottom) of Ly5.1^+^ OT‐II cells normalized to 1 × 10^5^ PBMCs. (f) Kinetic graphs showing dynamics in the frequencies and cell numbers of Ly5.1^+^ OT‐I and OT‐II cells over time. Black arrows on the graphs indicate the boosting time point: Day 21. (g,h) Ki67 expression in Ly5.1^+^ OT‐I cells within PBMC was analyzed by flow cytometry. (g) Concatenated flow plots showing the frequencies of Ki67^+^ CD8^+^ T cells in PBMC on days 8, 26, and 42. The grey dots represented the total CD8^+^ T cells, and the purple dots represented Ly5.1^+^ OT‐I cells in PBMC. (h) Graphs showing the frequencies (top) and cell numbers (bottom) of Ki67^+^ OT‐I cells per 1 × 10^5^ PBMCs. The data are representative of two independent experiments. Column graphs show mean ± SD, and kinetic graphs show mean ± SEM. Statistical significance was analyzed by one‐way ANOVA with Tukey's multiple comparisons test. ns, not statistically significant; ^*^ *p* < 0.05; ^**^ *p* <0.01; ^***^ *p* < 0.001; ^****^ *p* < 0.0001.

To analyze the antigen‐specific T cell responses induced by the ML‐DMN vaccine, we co‐transferred CD45.1 (Ly5.1^+^) OVA‐specific CD4^+^ (OT‐II) and CD8^+^ (OT‐I) T cells into CD45.2 (Ly5.2^+^) naïve mice (Figure 5a). The mRNA vaccines were administered on day 0 and boosted on day 21. Antigen‐specific T cells in mouse peripheral blood mononuclear cells (PBMC) and organs (spleen and lungs) were analyzed using flow cytometry (Figure 5a). On day 8, the ML‐DMN group exhibited a dramatic increase in OT‐I and OT‐II cells in the PBMC (Figure 5b–f). Similarly, after vaccine boosting on day 21, the ML‐DMN vaccine generated the highest frequency and number of OT‐I cells in PBMC on day 26 (Figure 5b,c,f). Furthermore, vaccination with ML‐DMN resulted in a superior boosting effect compared with other forms of mRNA vaccines, and antigen‐specific T cells induced by ML‐DMN persisted for a longer duration, indicating the potential of ML‐DMN to provide long‐term immunity (Figure 5b,c,f). The antigen‐specific CD4^+^ T cell response peaked on day 8 along with the antigen‐specific CD8^+^ T cell response, however, in contrast to the OT‐I cell response, it gradually decreased over time, showing no boosting effect, even after the second vaccination on day 21 (Figure 5d–f). Nevertheless, the frequency and number of OT‐II cells were still the highest in ML‐DMN‐vaccinated mice, even on day 26 (Figure 5d,e).

The weaker CD4^+^ T cell response compared to that of CD8^+^ T cells arose from differences in priming mechanisms. Unlike CD8^+^ T cells, which are primed by intracellular antigens presented to MHC class I molecules, priming of CD4^+^ T cells requires the uptake of extracellular (or cell surface) antigens by dendritic cells, leading to antigen presentation to MHC class II molecules [56, 57]. Although OVA mRNA vaccines can generate both endogenous (MHC class I) and exogenous antigens (MHC class II) through the ER‐Golgi pathway, the weaker CD4^+^ T cell response possibly resulted from the antigenic properties of OVA, which led to inefficient uptake by APC [58]. OVA, a major egg white protein, lacks pathogen‐associated molecular patterns (PAMPs), which facilitate antigen uptake by APCs through pattern recognition receptors (PRRs) such as C‐type lectins and toll‐like receptors (TLRs) [59, 60, 61, 62]. This reduced internalization of OVA antigens likely limited their presentation on MHC class II molecules, resulting in minimal CD4^+^ T cell activation, despite the ability of OVA mRNA to produce secreted antigens in the exogenous environment.

To determine whether the observed increase in OT‐I and OT‐II cells in PBMCs resulted from cellular proliferation, we analyzed the expression of Ki67, a proliferation marker, in these antigen‐specific T cells using flow cytometry. ML‐DMN vaccination significantly increased the Ki67^+^ OT‐I cell frequency and number, with continuously high expression observed on days 8, 26, and 42 post‐boost, marking the highest levels among all groups (Figure 5g,h).

This PBMC activation reflected the induction of systemic immune responses, with ML‐DMN inducing substantial antigen‐specific CD8^+^ T cell activation through enhanced proliferation, a hallmark of effective cell‐mediated immunity. Furthermore, ML‐DMN vaccination showed a superior booster effect compared to conventional IM injections, sustaining long‐term T cell proliferation over time. By cultivating cell‐mediated immune responses, which are critical for combating intracellular pathogens and ensuring lasting immune protection, ML‐DMN emphasized its ability to support the development of durable and resilient adaptive immune memory.

### 2.7 In Vivo Assessment of Tissue‐Specific Adaptive Immune Responses in Spleen and Lung through Antigen‐Specific T Cell Activity

**FIGURE 6:**
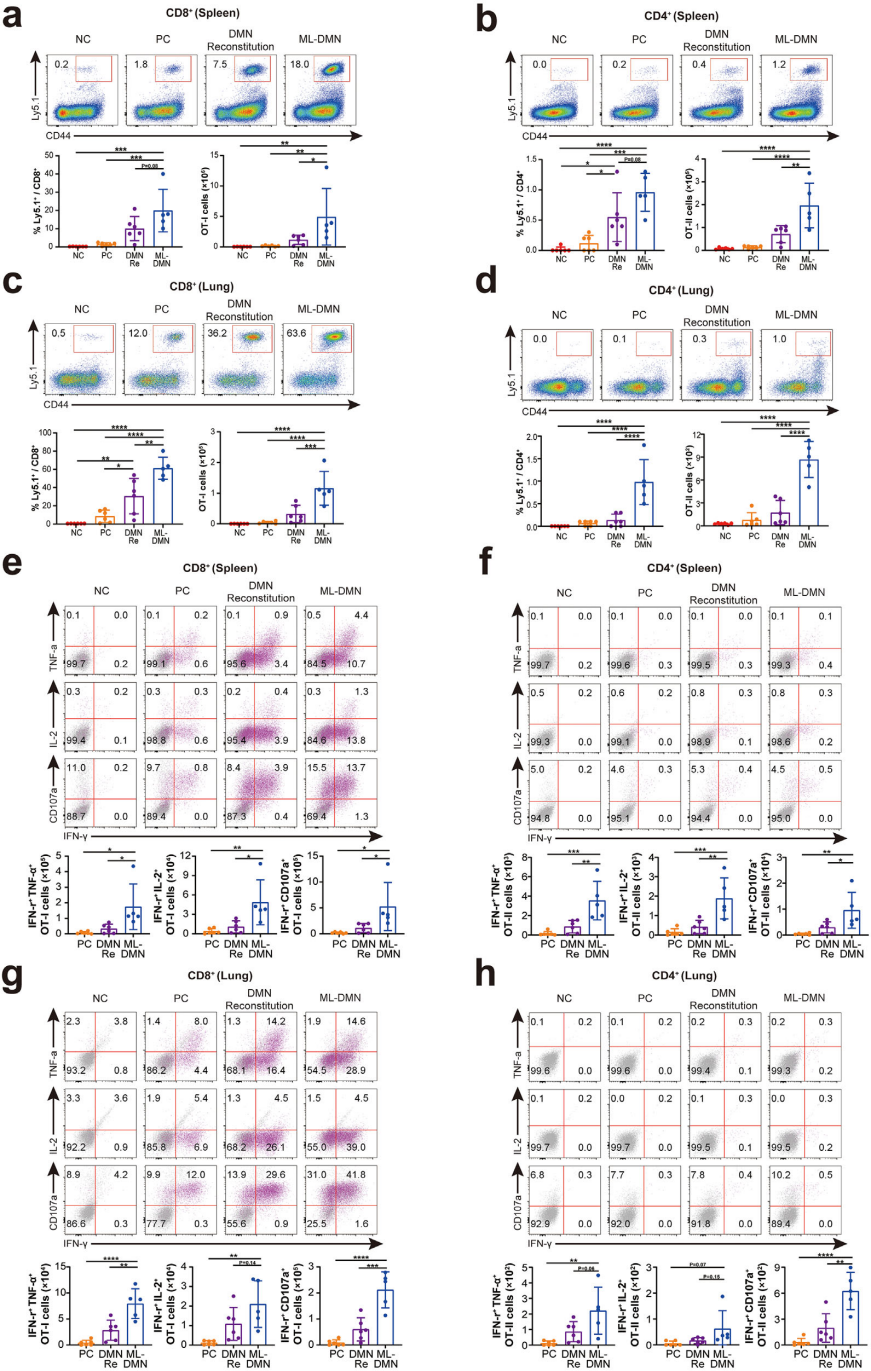
Analysis of antigen‐specific T cells in the spleen and lung of mice vaccinated with 1.5 µg OVA mRNA vaccines assessed by flow cytometry (n = 5–6 per group). (a,b) Representative flow plots and graphs depicting the frequencies and absolute numbers of (a) Ly5.1^+^ OT‐I and (b) Ly5.1^+^ OT‐II cells in the spleen, gated on CD8^+^ and CD4^+^ T cells, respectively. (c,d) Representative flow plots and graphs depicting the frequencies and absolute number of (c) Ly5.1^+^ OT‐I and (d) Ly5.1^+^ OT‐II cells in the lung, gated on CD8^+^ and CD4^+^ T cells. (e–h) The effector function of antigen‐specific T cells in the mouse (e,f) spleen and (g,h) lung was assessed. For cytokine production analysis, immune cells were stimulated with 0.2 µg/mL OVA_257‐264_ and 5 µg/mL OVA_323‐339_ for 6 h at 37°C, followed by flow cytometry. (e,f) Representative flow plots showing the frequencies of cytokine‐ (IFN‐γ^+^, TNF‐α^+^, IL‐2^+^, CD107a^+^) and multi‐cytokine‐ (IFN‐γ^+^ TNF‐α^+^, IFN‐γ^+^ IL‐2^+^, IFN‐γ^+^ CD107a^+^) producing (e) Ly5.1^+^ OT‐I and (f) Ly5.1^+^ OT‐II cells in the spleen. (g,h) Representative flow plots showing the frequencies of cytokine‐ (IFN‐γ^+^, TNF‐α^+^, IL‐2^+^, CD107a^+^) and multi‐cytokine‐ (IFN‐γ^+^ TNF‐α^+^, IFN‐γ^+^ IL‐2^+^, IFN‐γ^+^ CD107a^+^) producing (g) Ly5.1^+^ OT‐I and (h) Ly5.1^+^ OT‐II cells in the lung. In all plots, grey dots indicate total CD8^+^ or CD4^+^ T cells, and purple dots represent Ly5.1^+^ antigen‐specific cells. Bar graphs show absolute numbers of multi‐cytokine‐producing cells in each group. The data are representative of two independent experiments. Column graphs show mean ± SD. Statistical significance was analyzed by one‐way ANOVA with Tukey's multiple comparisons test. ns, not statistically significant; ^*^ *p* < 0.05; ^**^ *p* < 0.01; ^***^ *p* < 0.001; ^****^ *p* < 0.0001.

To comprehensively evaluate the immune responses induced by ML‐DMN vaccination, we extended our analysis to T cell activation in lymphoid tissues such as the spleen, which is essential for initiating systemic immune responses, and non‐lymphoid sites such as the lungs, which serve as critical tissues in localized immune protection. We analyzed these complementary immune compartments in the NC, PC, DMN reconstitution, and ML‐DMN groups on day 27 post‐vaccination (Figure 5a). Similar to the patterns previously observed in PBMC, the frequency and number of OT‐I and OT‐II cells in the spleen were significantly higher in the ML‐DMN‐vaccinated mice (Figure 6a,b). Compared to the PC group, the ML‐DMN group exhibited a massive increase of approximately 6‐ to 10‐fold in the frequency and number of antigen‐specific T cells (Figure 6a,b). ML‐DMN amplified antigen‐specific T cell responses, driving systemic immune activation and building a robust foundation for defense against future pathogen exposure.

Next, to evaluate the protective effect of ML‐DMN in non‐lymphoid organs, antigen‐specific T cells in the lungs were analyzed, which revealed a dramatic increase in the frequency and number of OT‐I and OT‐II cells (Figure 6c,d). The frequency and number of antigen‐specific T cells were substantially increased in the ML‐DMN group by approximately 6‐ to 10‐fold compared to the liposome‐vaccinated PC group, and 2‐ to 4‐fold compared to the DMN reconstitution group (Figure 6c,d). These results revealed that ML‐DMN vaccination efficiently recruited and activated T cells in peripheral tissues, providing robust localized immune protection against infections at primary entry sites, such as the respiratory tract.

To further investigate whether vaccination through ML‐DMN could also change the functionality of vaccine‐induced antigen‐specific T cells, OT‐I and OT‐II cells from mouse organs were re‐stimulated with OVA_257‐264_ and OVA_323‐339_ peptides and analyzed by flow cytometry for cytokine production. ML‐DMN vaccination resulted in a significant increase in the frequency and number of cytokine‐producing OT‐I and OT‐II cells in the spleen (Figure 6e,f; Figure S3). Specifically, the ML‐DMN group exhibited significantly higher frequencies and numbers of cytokine‐producing OT‐I cells, including subsets producing multiple cytokines such as IFN‐γ, TNF‐α, IL‐2, and CD107a, compared to other groups (Figure 6e; Figure S3a,b). Similarly, ML‐DMN vaccination greatly enhanced the generation of cytokine‐ or multi‐cytokine‐producing OT‐II cells (Figure 6f; Figure S3c,d). Thus, these data indicated that ML‐DMN vaccines improved the effector function of antigen‐specific T cells in the spleen, supporting effective pathogen clearance and the establishment of immune memory.

As mentioned above, cytokine and CD107a expression in OT‐I and OT‐II cells in the lungs was assessed, revealing a significant increase in the frequency and number of cytokine‐ or multi‐cytokine‐producing OT‐I cells in the ML‐DMN group, similar to that in the spleen (Figure 6g; Figure S4a,b). Likewise, ML‐DMN vaccines also generated more IFN‐γ^+^, TNF‐α^+^, IL‐2^+^, and CD107a^+^ OT‐II cells (Figure 6h; Figure S4c,d). These findings revealed that ML‐DMN vaccines extended their immunogenic effects from central lymphoid tissues to peripheral sites prone to infection. Taken together, ML‐DMN vaccination enhanced the effector function of antigen‐specific T cells in both the spleen and lungs, combining robust systemic responses with targeted protection in infection‐prone tissues to ensure a multifaceted immune defense. This capability underscored the versatility of ML‐DMN in triggering adaptive immune responses tailored for comprehensive and effective protection.

### 2.8 Evaluation of Dose‐Sparing Effect of ML‐DMN Vaccines for T Cell Immune Response

Expanding upon the robust innate and adaptive immune responses observed at the same 1.5 µg dose, our findings indicated that this dose was sufficient to elicit strong immune activation, contrasting with other studies utilizing higher doses of 5–10 µg [29, 36, 37]. To investigate whether this observed efficacy reflects the dose‐sparing potential of ML‐DMN, we evaluated its performance at a higher dose of 3 µg, aiming to confirm its capacity for elevated immune activation compared to IM injection. Six experimental groups (NC, Blank DMN, PC, DMN solution, DMN reconstitution, and ML‐DMN) receiving 3 µg of OVA mRNA were established to distinguish matrix‐, formulation‐, and fabrication‐dependent effects on immune activation (Figures S5 and S6). The Blank DMN group, composed of the same polymeric matrix and excipient components without mRNA, was included to verify that the microneedle matrix and excipients themselves did not induce antigen‐specific T cell responses, while the DMN solution group was used to examine the impact of fabrication processes on mRNA efficacy. Using the same experimental scheme shown in Figure 5a, ML‐DMN consistently showed superior efficacy, including enhanced boosting effects and prolonged antigen‐specific T cell responses, as indicated by sustained Ki67 expression up to day 42 post‐vaccination (Figure S5). Furthermore, ML‐DMN vaccination markedly increased the frequencies and numbers of OT‐I and OT‐II cells in the spleen and lungs (Figure S6). Consistent with the NC group, Blank DMN did not result in measurable expansion of OT‐I or OT‐II cells, confirming that immune activation was attributable to mRNA delivery rather than the polymeric DMN matrix itself.

**FIGURE 7:**
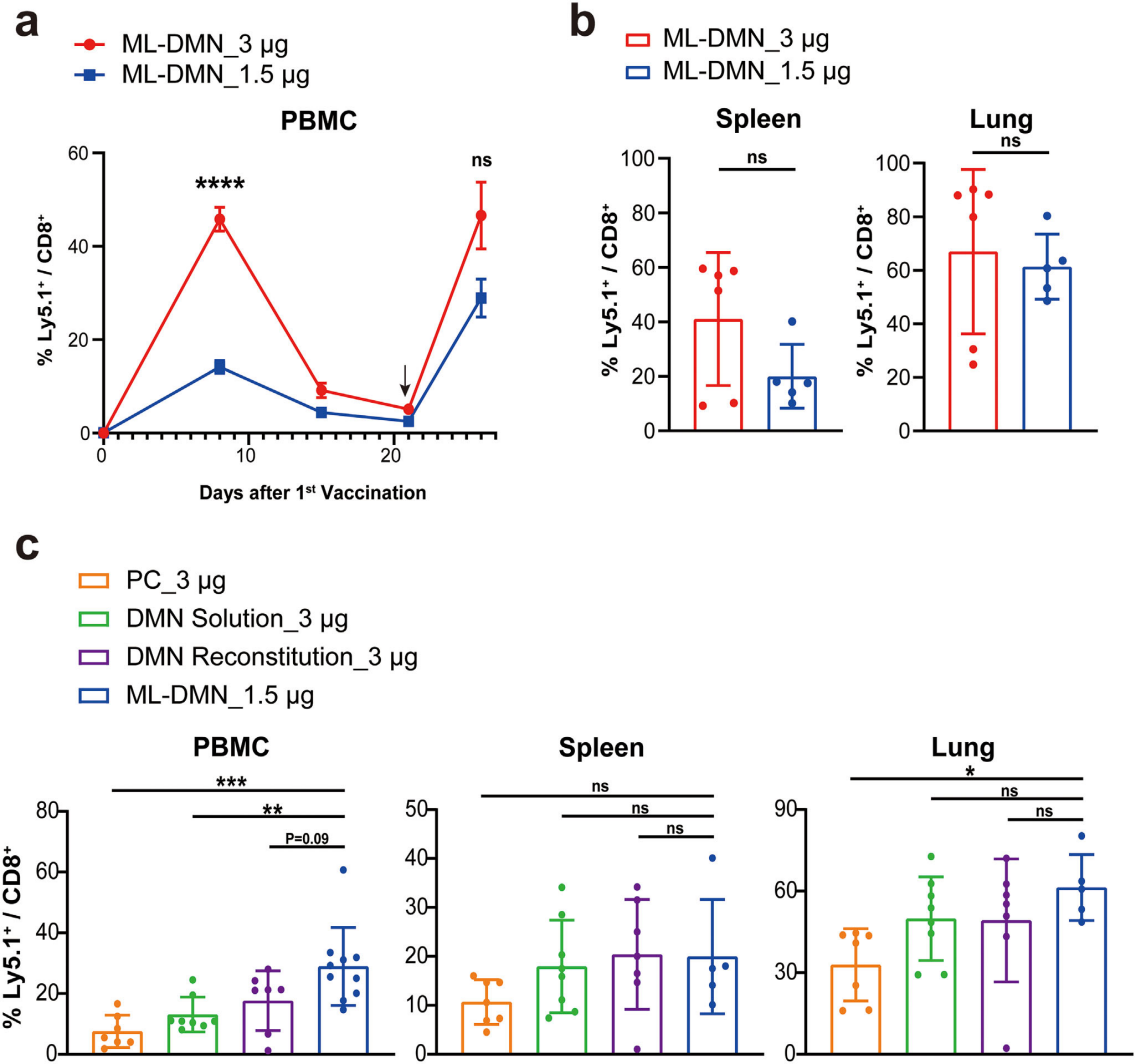
Evaluation of Ly5.1^+^ OT‐I cell dynamics from mice immunized with ML‐DMN vaccines containing 1.5 or 3 µg OVA mRNA. (a) Kinetics graph showing dynamics of Ly5.1^+^ OT‐I cell frequency in PBMC of mice immunized with ML‐DMN vaccines loaded with 3 µg (n = 6) vs. 1.5 µg (n = 5) OVA mRNA. The black arrow on the graph indicates the boosting time point: Day 21. (b) Graphs showing the frequencies of Ly5.1^+^ OT‐I cells present in the spleen (left) and lung (right) of mice immunized with ML‐DMN vaccines loaded with 3 µg (n = 6) vs. 1.5 µg (n = 5) OVA mRNA. (c) Graphs showing the frequencies of Ly5.1^+^ OT‐I cells present in day 26 PBMC (left), spleen (middle), and lung (right) of mice immunized with various forms of mRNA vaccines loaded with 3 µg OVA mRNA or ML‐DMN vaccines loaded with 1.5 µg OVA mRNA. PC, DMN solution, and DMN reconstitution group received the vaccines made with the same composition and state as described above, but loaded with 3 µg mRNA. The data are representative of two independent experiments. The kinetics graph shows mean ± SEM, and column graphs show mean ± SD. Statistical significance was analyzed by two‐way ANOVA using Geisser–Greenhouse correction with Sidak's multiple comparisons test in (a), by two‐tailed unpaired Student's *t*‐test in (b), and by one‐way ANOVA with Tukey's multiple comparisons test in (c). ns, not statistically significant; ^*^ *p* < 0.05; ^**^ *p* < 0.01; ^***^ *p* < 0.001; ^****^ *p* < 0.0001.

Despite the higher dose of 3 µg mRNA, the observed trends remained consistent, illustrating the dose‐sparing effect of ML‐DMN vaccination. For example, the kinetics graph of OT‐I cells in peripheral blood showed that ML‐DMN with 3 µg elicited more antigen‐specific T cells than the 1.5 µg group on day 8. (Figure 7a). However, following the second vaccination on day 21, the 1.5 µg ML‐DMN group exhibited a sharper increase in OT‐I, reaching a comparable frequency to the 3 µg group by day 26 (Figure 7a). This cross‐dose comparison demonstrated that a lower dose of ML‐DMN could achieve immune responses comparable to or exceeding those induced by a doubled IM dose. Similarly, on day 27, the frequency of OT‐I cells in the spleen and lung showed no significant differences between 3 and 1.5 µg mRNA vaccination through ML‐DMN (Figure 7b). Furthermore, ML‐DMN vaccination with 1.5 µg mRNA elicited a similar or even stronger antigen‐specific T cell response compared to the other injection groups loaded with a doubled dose (3 µg) of mRNA, highlighting its higher efficacy at lower doses (Figure 7c).

Prior MN‐based mRNA vaccine studies demonstrated improved formulation stability and effective immune responses using transdermal platforms [37, 38, 39]. However, systematic evaluation of dose‐sparing effects at the level of antigen‐specific T cell expansion under identical experimental conditions was less explored. In contrast, we employed a unified experimental framework with matched controls to assess the dose‐sparing capacity of ML‐DMN and its impact on booster durability, thereby clarifying its functional immune efficiency. These findings provided significant implications for vaccine development. Reducing the required mRNA dose could lower vaccine production costs and increase accessibility, especially in contexts requiring large‐scale distribution. Moreover, the enhanced boosting effect at lower doses may help minimize the potential side effects associated with higher mRNA quantities, further supporting the use of ML‐DMN as an efficient delivery system for mRNA vaccines.

### 2.9 Analysis of Anti‐OVA IgG Antibody Responses

**FIGURE 8:**
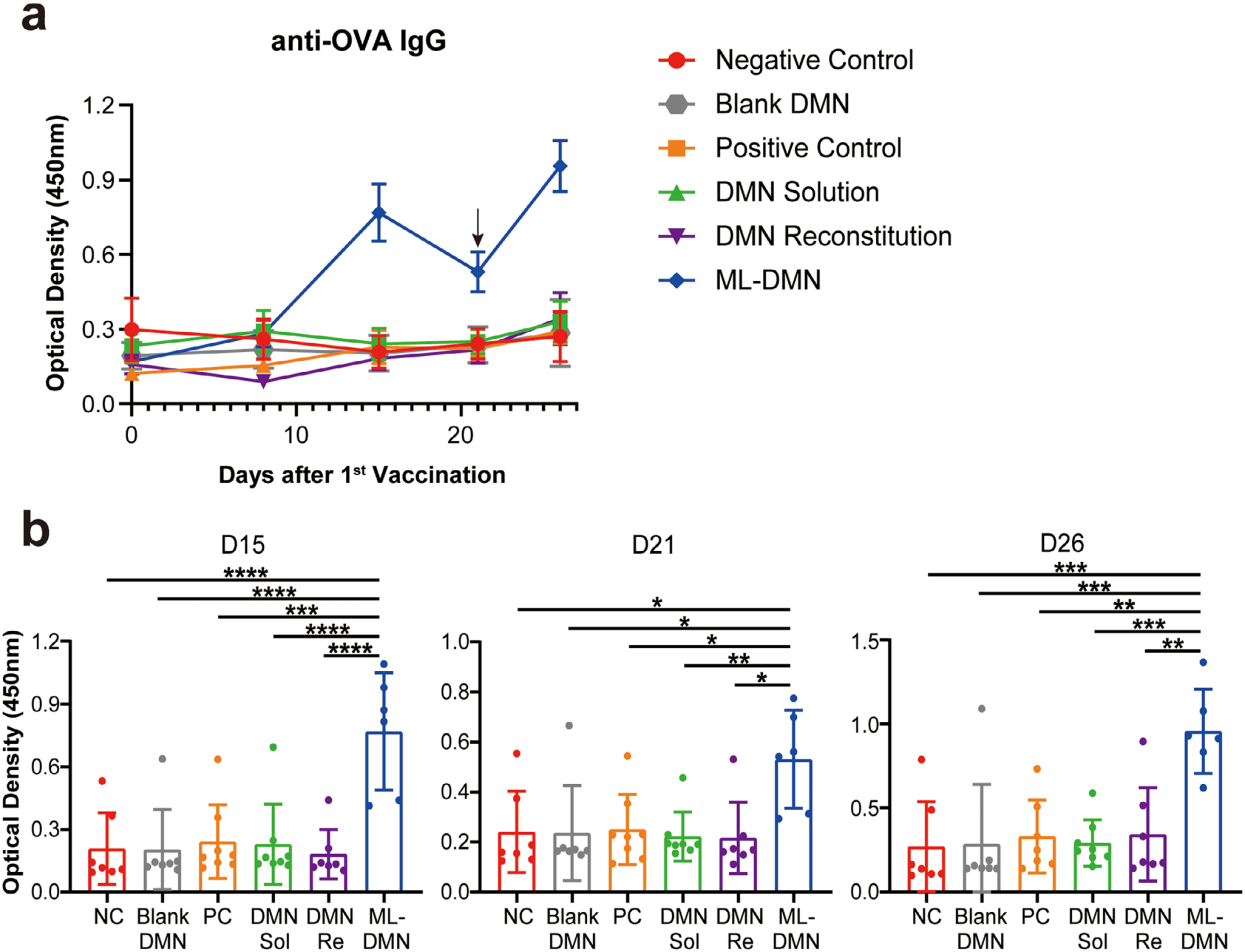
Amount of anti‐OVA IgG antibody in serum from the mice immunized with 3 µg OVA mRNA was analyzed by ELISA (n = 6‐8 per group). ELISA plates coated with 10 µg/mL of full‐length OVA protein were incubated with diluted serum (100×), and the optical density was measured at 450 nm. (a) Kinetic graph showing the dynamics of anti‐OVA IgG antibody after 1st vaccination. The black arrow on the graph indicates the boosting time point: Day 21. (b) Graphs showing the relative amount of anti‐OVA IgG antibody in mouse serum at the indicated time points. The data are representative of two independent experiments. Column graphs show mean ± SD, and the kinetics graph shows mean ± SEM. Statistical significance was analyzed by one‐way ANOVA with Tukey's multiple comparisons test. ns, not statistically significant; ^*^ *p* < 0.05; ^**^ *p* < 0.01; ^***^ *p* < 0.001; ^****^ *p* < 0.0001.

ML‐DMN promoted efficient antigen‐specific T cell responses, even at a low mRNA dose of 1.5 µg, outperforming conventional mRNA vaccines. Although these results underscore the efficacy of ML‐DMN in driving cell‐mediated immunity, the full potential for vaccine‐induced protection requires a coordinated humoral immune response to ensure effective pathogen neutralization and long‐term immunity. To further examine the scope of ML‐DMN's immunogenicity, we analyzed antibody production in mice immunized with OVA mRNA at doses of 1.5 and 3 µg (Figure 8; Figure S7). At a 1.5 µg dose of ML‐DMN vaccination, unlike strong antigen‐specific T cell responses, antibody generation remained undetectable (Figure S7).

At the higher dose of 3 µg, ML‐DMN successfully elicited a significant antibody response alongside enhanced T cell activation (Figure 8). The ML‐DMN group showed the highest IgG antibody levels in the mouse serum, with significantly increased anti‐OVA IgG production compared to the other injection groups, which exhibited no difference from the NC group (Figure 8a,b). To further evaluate immune polarization, IgG subclass analysis was performed by measuring serum IgG1 and IgG2c levels, which are commonly used surrogate markers for Th2‐ and Th1‐associated humoral responses, respectively, in mouse models. The results demonstrated that ML‐DMN vaccination induced robust IgG2c responses together with detectable IgG1 responses (Figure S8), indicating the presence of both Th1‐ and Th2‐associated humoral immunity. These findings suggested that ML‐DMN vaccination induced a predominantly Th1‐associated immune response while maintaining measurable Th2‐associated antibody production. Antibody responses were evaluated based on optical density values obtained under strictly identical ELISA conditions across all experimental groups, enabling direct relative comparison of antigen‐specific IgG levels. Statistical analysis confirmed that no significant difference was observed between the NC and PC groups, whereas ML‐DMN at 3 µg induced significantly higher IgG responses compared with all injection‐based groups. This dose‐dependent antibody response suggested that with an optimized mRNA dose, ML‐DMN could effectively engage CD4^+^ T cells to support both cellular and humoral immunity.

These observations were interpreted in the context of previously reported immune responses induced by clinically validated mRNA–LNP vaccines, which have demonstrated robust induction of both CD4^+^ T cell responses and antibody production following IM administration in both clinical and preclinical studies [54, 63, 64]. These studies collectively highlighted that the magnitude of humoral immune responses was influenced by multiple factors, including antigen immunogenicity, delivery formulation, and administered mRNA dose. In contrast to viral antigens such as SARS‐CoV‐2 spike protein, which inherently contain structural and molecular features facilitating recognition of APC, OVA represents a relatively weak model antigen. Therefore, the limited antibody responses observed in injection‐based control groups in this study might reflect antigen‐dependent immune activation thresholds rather than intrinsic limitations of injection‐based mRNA vaccination platforms. Under these experimental conditions, ML‐DMN delivery enabled detectable antibody induction at higher mRNA doses, suggesting that transdermal delivery through immune cell‐rich skin microenvironments might enhance antigen presentation efficiency and downstream CD4^+^ T cell‐dependent humoral immune activation.

Although the limitations of OVA as a less immunogenic model antigen could not reflect the full potential of ML‐DMN in real‐world applications, ML‐DMN vaccination activated an adaptive immune response even under suboptimal conditions, underlining its promise as a versatile mRNA vaccine delivery system. Compared with conventional methods, ML‐DMN applications achieved potent T cell responses at the same mRNA dose, whereas an equivalent dose was required for a robust antibody response, demonstrating their practicality for scalable vaccine strategies. This approach could optimize vaccine regimens by reducing antigen input without compromising long‐term immunity. By effectively engaging in both cell‐mediated and humoral immunity, the ML‐DMN established itself as a strong candidate for mRNA vaccine delivery systems capable of balancing immune responses based on antigen properties and optimizing vaccine efficacy across different immunological contexts.

## 3 Conclusions

In this study, the ML‐DMN vaccines effectively addressed the critical challenges of mRNA vaccine delivery, including potency, immune activation, and dose efficiency. Optimization of the formulation with stabilizing agents, such as trehalose and sucrose, preserved mRNA functionality during fabrication, as demonstrated by both in vitro and in vivo luciferase assays, confirming sustained activity and incorporation. ML‐DMN systems initiated robust innate immune responses marked by the rapid activation and migration of APCs to dLNs. This led to potent adaptive immunity, characterized by significant proliferation of antigen‐specific T cells and production of multifunctional cytokines, highlighting the ability of ML‐DMN vaccines to drive effective cell‐mediated immunity, even at lower mRNA doses than traditionally required. Additionally, ML‐DMN exhibited a dose‐dependent immunogenic profile, inducing strong T cell responses across different mRNA doses. This platform also elicited substantial antibody production at an equivalent mRNA dose to injection, amplifying both cell‐mediated and humoral immunity, which is critical for comprehensive vaccine‐generated protection beyond that achieved by injections. Although this study employed OVA as a model antigen, further research using clinically relevant antigens is required to fully explore the potential of ML‐DMN systems. These findings established ML‐DMN as a future mRNA vaccination, offering improved potency, logistical advantages, and flexibility to adapt to diverse immunological needs.

## 4 Experimental Section

### 4.1 Materials

Unmodified firefly luciferase mRNA and OVA mRNA were obtained from OZ Biosciences (San Diego, CA, USA). In vivo‐jetRNA+ liposome reagents were purchased from Polyplus (Illkirch, France). HA (32 kDa) was sourced from Uscare Pharm (Shanghai, China). Trehalose, sucrose, calcein, rhodamine B, D‐luciferin, and tetramethylbenzidine (TMB) solution were from Sigma–Aldrich (St. Louis, MO, USA). Full‐length OVA protein was obtained from Sigma–Aldrich, and bovine serum albumin (BSA) from Santa Cruz Biotechnology (Dallas, TX, USA). HRP‐conjugated anti‐mouse IgG was from GenDEPOT (Katy, TX, USA). The J774A.1 murine macrophage cell line (RRID: CVCL_0358) was purchased from the American Type Culture Collection (ATCC, Manassas, VA, USA) in August 2023, authenticated by the supplier, and confirmed to be free from mycoplasma contamination prior to use. Porcine cadaver skin was purchased from CRONEX (Hwaseong, South Korea). Mouse CD4^+^ and CD8a^+^ T Cell Isolation Kits were from Miltenyi Biotec (Bergisch Gladbach, Germany). Live/Dead Fixable Near‐IR Dead Cell Stain Kit was from Invitrogen (Waltham, MA, USA). GolgiPlug, GolgiStop, and Cytofix/Cytoperm Fixation/Permeabilization Kit were from BD Biosciences (San Jose, CA, USA).

### 4.2 Preparation of mRNA‐Liposome Formulations and Experimental Groups

Unmodified firefly luciferase mRNA (1 µg) and OVA mRNA (1.5 or 3 µg) were encapsulated in liposomes prepared using an in vivo‐jetRNA+ reagent according to the manufacturer's protocol. Diluted mRNA was complexed with in vivo‐jetRNA+ reagent at a mass‐to‐volume ratio of 1:2 (µg mRNA:µL reagent), followed by gentle pipetting to ensure homogeneous mixing. The mixture was incubated for 15 min at room temperature to allow self‐assembly of mRNA‐liposome complexes. The resulting mRNA‐liposomes, corresponding to 66.7% (v/v) of the final formulation, were subsequently mixed with 30% (w/v) HA solution, 15% (w/v) trehalose, and 5% (w/v) sucrose to generate a DMN solution for ML‐DMN fabrication or direct injection. The study used the following experimental groups: (a) NC (PBS), (b) Blank DMN, (c) PC (mRNA‐liposomes), (d) DMN solution, (e) ML‐DMN (DMN patches), and (f) DMN reconstitution (dissolved ML‐DMN).

### 4.3 Fabrication of ML‐DMN

ML‐DMN was fabricated using a sequential layering process that involved the creation of base, core, and shell layers, followed by shaping using centrifugal lithography, as previously described for multilayer dissolving microneedle fabrication and centrifugal shaping techniques [43, 44, 46]. A 65% (w/v) HA solution was dispensed using ML‐5000X (Musashi Engineering, Tokyo, Japan), followed by the DMN core solution and a 60% (w/v) HA shell. Shaping was performed at 200 × *g* for 1 min to shape the viscous solution into its final microneedle form.

### 4.4 Assessment of the Structure and Mechanical Integrity of ML‐DMN

ML‐DMN was stained with calcein (green) and rhodamine B (orange), and examined using confocal laser scanning microscopy (Confocal; LSM980, Carl Zeiss, Oberkochen, Germany). The mechanical integrity of each DMN was evaluated using a displacement force device (Z0.5TN; Zwick/Roell, Ulm, Germany). A single DMN was separated from the ML‐DMN patch, securely positioned on a custom‐made patch substrate, and subjected to a gradually lowered sensor probe at a constant speed of 1.0 mm/min until contact was achieved.

### 4.5 Ex Vivo Skin Insertion Test

To observe skin penetration, porcine cadaver skin was rinsed with 1× PBS and air‐dried for 15 min to remove moisture. The ML‐DMN patch was applied vertically to the thawed skin using a custom‐made applicator. After 15 min of application, skin penetration and insertion were confirmed using M165FC fluorescence microscopy (Leica, Wetzlar, Germany) and OCT (LabScope 2.0 OCT, Lumedica, Durham, NC, USA).

### 4.6 In Vitro Luciferase Assay

J774A.1 cells were seeded at a density of 1.5 × 10^5^ cells/well in 24‐well plates (SPL Life Sciences, Pocheon‐si, South Korea). Each experimental group received 0.5 µg of firefly luciferase mRNA: (a) NC (PBS), (b) PC (luciferase mRNA‐liposomes), (c) HA solution (mRNA‐liposomes mixed with 30% (w/v) HA without drying), (d) HA‐DMN (mRNA‐liposomes mixed with 30% (w/v) HA solution and subjected to the drying and fabrication processes), and (e) formulations with varying concentrations of trehalose and sucrose (0%, 5%, 10%, 15%, 20% (w/v)) incorporated into HA solutions, followed by the fabrication processes. Luminescence was analyzed after 24 h using the Luciferase Assay System (Promega, Madison, WI, USA) with a luminometer (LUMIstar Omega Microplate Reader, BMG Labtech, Ortenberg, Germany).

### 4.7 Characterization of mRNA‐Liposomes

The size of the mRNA‐liposomes was measured using DLS with a Particle Size & Zeta Potential Analyzer (ELS‐Z1000, Otsuka Electronics, Osaka, Japan). Morphology of mRNA‐liposomes (2 µg mRNA/sample) was examined using TEM (JEM‐F200, JEOL, Tokyo, Japan) after staining with 1% phosphotungstic acid.

### 4.8 IVIS Imaging

In vivo expression of luciferase mRNA was assessed using an IVIS Spectrum (PerkinElmer, Waltham, MA, USA). BALB/c mice (6–8 weeks old) were used for luciferase imaging to minimize optical attenuation of bioluminescent signals by skin pigmentation, as melanin has been reported to reduce signal intensity in optical imaging studies [65]. Mice were randomly assigned to five groups: (a) NC, (b) PC, (c) DMN solution, (d) DMN reconstitution, and (e) ML‐DMN. Each group received 1 µg of firefly luciferase mRNA using ID injection or ML‐DMN application (84‐needle array for 1 µg). The mRNA concentration used for in vivo luciferase imaging was fixed based on prior in vitro optimization. The formulation composition and per‐needle mRNA loading were identical across experiments, and the total dose was adjusted by scaling the number of needles per array. At 6, 12, 24, and 48 h post‐administration, mice were intraperitoneally injected with D‐luciferin (150 mg/kg) 10 min prior to imaging. Luminescence was recorded using Living Image software (PerkinElmer, Waltham, MA, USA) to evaluate mRNA expression and distribution. All other experimental parameters relevant to IVIS analysis, such as administered dose, imaging time points, acquisition settings, and Region of Interest (ROI) definition, were kept identical across groups. Given that bioluminescent signal intensity can be influenced by anatomical and physiological factors, including tissue thickness, optical attenuation, and local blood flow, IVIS measurements were interpreted primarily in terms of relative expression kinetics and temporal persistence rather than absolute signal intensity.

### 4.9 mRNA Vaccination of Innate Immune Responses

To analyze innate immune response at an early time point after vaccination, wild‐type C57BL/6 mice were immunized with OVA mRNA (1.5 µg) using ID injection or ML‐DMN (126‐needle array, scaled to maintain constant per‐needle mRNA loading while achieving a total dose of 1.5 µg), corresponding to experimental groups: (a) NC, (b) PC, (d) DMN reconstitution, and (e) ML‐DMN. All vaccines were administered to the dorsal skin to match the location of the dLNs (inguinal LNs). All immunological analyses were conducted under fully standardized conditions, including consistent mouse strain, matched administration sites, and controlled experimental parameters, to ensure reliable comparison of immune responses. At 24 h after vaccination, the mice were sacrificed, and dLNs were harvested for flow cytometric analysis. The vaccination sites, time points of analysis, and location of dLNs were determined based on the previous research [30, 54, 55].

### 4.10 Adoptive Transfer of OVA‐Specific T Cells and mRNA Vaccination for Observation of Adaptive Immune Responses

C57BL/6 mice were obtained from Orient Bio (Seongnam, Korea) at eight weeks of age. TCR‐transgenic OT‐I Ly5.1 and OT‐II Ly5.1 mice were purchased from Jackson Laboratory (Bar Harbor, ME, USA). C57BL/6 mice were used for immunogenicity and adoptive transfer experiments because OT‐I and OT‐II TCR‐transgenic lines are maintained on a C57BL/6 (H‐2^b^) genetic background and require MHC‐matched antigen presentation for accurate evaluation of antigen‐specific CD8^+^ and CD4^+^ T cell responses [66, 67]. Thus, different mouse strains were intentionally employed according to the specific experimental objectives, with BALB/c mice used to optimize optical imaging performance and C57BL/6 mice used to ensure immunological relevance and compatibility with TCR‐transgenic systems. For adoptive co‐transfer of OVA‐specific OT‐I (CD8^+^) and OT‐II (CD4^+^) cells, the spleens of OT‐I Ly5.1 and OT‐II Ly5.1 mice were harvested, and OT‐I (CD8^+^) and OT‐II (CD4^+^) cells were sorted from splenocytes by negative selection using a Mouse CD8a^+^ or CD4^+^ T cell Isolation Kit. After purification, 2 × 10^5^ OT‐I and 5 × 10^5^ OT‐II cells were co‐transferred into naïve Ly5.2^+^ mice (C57BL/6) via intravenous injection. One day post‐transfer, recipient mice were immunized with OVA mRNA (1.5 or 3 µg) using IM injection or ML‐DMN (126‐needle array for 1.5 µg and 252‐needle array for 3 µg, with identical formulation composition and fixed mRNA loading per needle; total dose controlled by array scaling), corresponding to experimental groups: (a) NC, (b) Blank DMN, (c) PC, (d) DMN solution, (e) DMN reconstitution, and (f) ML‐DMN. Booster immunization was administered 21 days after the first vaccination. The adoptive transfer of OVA‐specific T cells and vaccination time points were determined based on the previous research [68].

### 4.11 Flow Cytometry and Antibodies

Cells were stained for surface and intracellular markers and analyzed using CytoFLEX or CytoFLEX LX (Beckman Coulter, Brea, CA, USA). Data were processed with FlowJo software (Tree Star, Ashland, OR, USA). Detailed antibody information is provided in Table S1.

### 4.12 ELISA for Anti‐OVA IgG Antibody Detection

The amount of anti‐OVA IgG antibody in serum from the mice vaccinated with 3 µg OVA mRNA was analyzed by ELISA as previously described [68]. In detail, ELISA plates coated with 10 µg/mL of full‐length OVA protein (Sigma–Aldrich, St. Louis, MO, USA) were blocked with bovine serum albumin (1%; Santa Cruz Biotechnology, Dallas, TX, USA) at 37°C for 1 h. Subsequently, the plates were washed, and diluted serum (100×) was added to each well, followed by incubation at 37°C for 2 h. After incubation, HRP‐conjugated anti‐mouse IgG (GenDEPOT, Katy, TX, USA) was added as a secondary antibody, and the plates were again incubated at 37°C for 1 h. The plates were then washed, and TMB solution (tetramethylbenzidine solution; BioLegend, San Diego, CA, US) was added to each well, followed by incubation at 25°C for 5 min. Finally, the optical density of each well was measured at 450 nm using an automatic ELISA plate reader.

### 4.13 Ethics Statement

All animal experiments followed the ethical guidelines of the Yonsei Laboratory Animal Research Center (YLARC). Maintenance of the mice and all mouse experiments were performed with the approval of the Institutional Animal Care and Use Committee (IACUC) at Yonsei University (permit number: IACUC‐A‐202311‐1761‐02). All in vivo experiments were conducted in accordance with the Korean Food and Drug Administration (KFDA) guidelines.

### 4.14 Statistical Analysis

Statistical significance was analyzed using Prism software version 8.0.2 (GraphPad). Differences between the two groups were assessed using a two‐tailed unpaired Student's *t*‐test. In three or more groups, differences were assessed with one‐way ANOVA with Tukey's or Dunnett's multiple comparison test. Differences in kinetic graphs were evaluated by two‐way ANOVA using Geisser‐Greenhouse correction with Tukey's or Sidak's multiple comparison test, whereas differences between experimental groups in endpoint measurements were analyzed using one‐way ANOVA followed by Dunnett's multiple comparison test. A *P*‐value < 0.05 was considered statistically significant.

## Conflicts of Interest

Hyungil Jung is a founder and shareholder of Juvic Inc. and has submitted patents that have been or may be licensed to the company, which develops microneedle‐based products. These potential conflicts of interest have been disclosed and are managed by Yonsei University. Beyond these disclosures, the authors have no additional affiliations or financial interests related to the subject matter of this manuscript.

## Supporting information




**Supporting File**: advs75116‐sup‐0001‐SuppMat.docx.

## Data Availability

The data that support the findings of this study are available from the corresponding author upon reasonable request.
