# Supplementary material for: Transdermal Delivery of an mRNA‐Liposome Vaccine via Dissolving Microneedle to Preserve Vaccine Activity and Enhance Immune Activation
Source: Adv Sci (Weinh). 2026 Apr 9;13(34):e22846. doi: 10.1002/advs.202522846 (PMC13285151; doi:10.1002/advs.202522846)
Supplement: Supplementary file 1 — Supporting File: advs75116‐sup‐0001‐SuppMat.docx. [file ADVS-13-e22846-s001.docx]

**Supporting Information**

Transdermal Delivery of an mRNA-liposome Vaccine via Dissolving Microneedle to Preserve Vaccine Activity and Enhance Immune Activation

Jeehye Nam^1^, JiWon Ahn^1^, Jiwoo Shin, Nahong Lee, Youjin Lee, Sung min Cho, Paul Kim, Geonwoo Kang, Sang-Jun Ha^*^ and Hyungil Jung^*^

J. Nam, J. Shin, H. Jung

Department of Biotechnology, Yonsei University

50, Yonsei-ro, Seoul, 03722, Republic of Korea

E-mail: hijung@yonsei.ac.kr

J. Ahn, N. Lee, S.-J. Ha

Department of Biochemistry, Yonsei University

50, Yonsei-ro, Seoul, 03722, Republic of Korea

E-mail: sjha@yonsei.ac.kr

Y. Lee, S. M. Cho

Department of Integrative Biotechnology, Yonsei University

85, Songdogwahak-ro, Inchon, 21983 Republic of Korea

P. Kim, G. Kang, H. Jung

JUVIC Inc.

272, Digital-ro, Seoul, 08389, Republic of Korea

^1^These two authors contributed equally to this study as first authors.

^*^These two authors contributed equally to this study as corresponding authors.

^*^Corresponding author: Hyungil Jung (hijung@yonsei.ac.kr), Sang-Jun Ha (sjha@yonsei.ac.kr)

**Table S1.** List of antibodies used for flow cytometry

| Marker | Clone | Fluorochrome | Supplier (City, Country) |
| --- | --- | --- | --- |
| CD4 | RM4-5 | BV785 | BioLegend (San Diego, CA, USA) |
| CD8a | 53-6.7 | Alexa Fluor 700 | BioLegend (San Diego, CA, USA) |
| KLRG1 | 2F1 | BV421 | BioLegend (San Diego, CA, USA) |
| CD44 | IM7 | BV510 | BioLegend (San Diego, CA, USA) |
| CD45.1 | A20 | APC | BioLegend (San Diego, CA, USA) |
| CD80 | 16-10A1 | BV421 | BioLegend (San Diego, CA, USA) |
| CD86 | GL-1 | FITC | BioLegend (San Diego, CA, USA) |
| CD11c | N418 | BV510 | BioLegend (San Diego, CA, USA) |
| CD103 | 2E7 | BV605 | BioLegend (San Diego, CA, USA) |
| I-A/I-E | M5/114.15.2 | BV785 | BioLegend (San Diego, CA, USA) |
| Siglec-H | 551 | APC | BioLegend (San Diego, CA, USA) |
| CD64 | X54-5/7.1 | APC | BioLegend (San Diego, CA, USA) |
| CD90.2 | 53-2.1 | PerCP-Cy5.5 | BioLegend (San Diego, CA, USA) |
| CD19 | 1D3/CD19 | PerCP-Cy5.5 | BioLegend (San Diego, CA, USA) |
| NK1.1 | PK136 | PerCP-Cy5.5 | BioLegend (San Diego, CA, USA) |
| CD107a | 1D4B | PerCP-Cy5.5 | BioLegend (San Diego, CA, USA) |
| Ki67 | 16A8 | PE-Dazzle594 | BioLegend (San Diego, CA, USA) |
| TNF-α | MP6-XT22 | BV421 | BioLegend (San Diego, CA, USA) |
| CD62L | MEL-14 | FITC | BD Biosciences (San Diego, CA, USA) |
| CD11b | M1/70 | PE-Cy7 | BD Biosciences (San Diego, CA, USA) |
| IFN-γ | XMG1.2 | FITC | BD Biosciences (San Diego, CA, USA) |
| IL-2 | JES6-5H4 | PE | BD Biosciences (San Diego, CA, USA) |
| F4/80 | BM8 | PE | eBioscience (San Diego, CA, USA) |

**
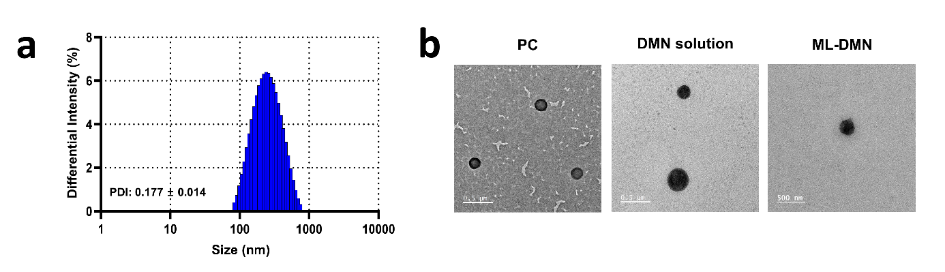
**

**Figure S1.** Structural integrity and characterization of mRNA-liposomes via DLS and TEM analysis. a) DLS analysis of mRNA-liposomes showing the particle size and PDI (n = 3, mean ± SEM). b) TEM images showing the structural integrity of the mRNA-liposomes: PC, DMN solution, and ML-DMN (scale bar, 500 nm).

**
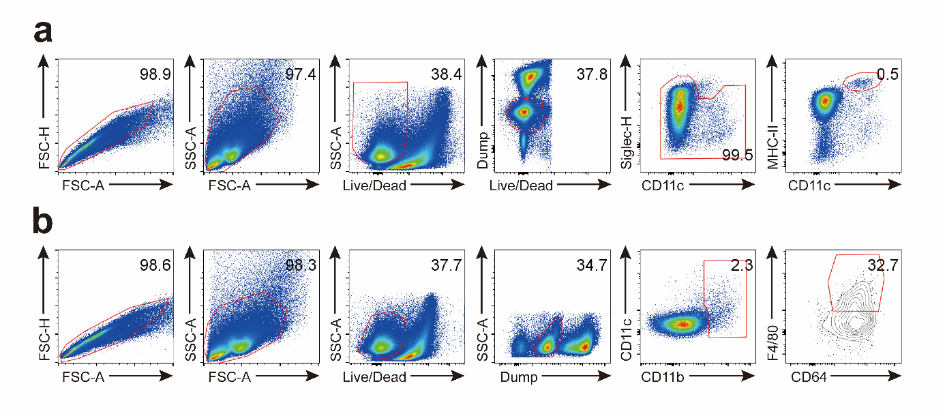
Figure S2.** Gating strategies used for flow cytometric analysis of a) cDCs and b) macrophages. ‘Dump’ indicates Thy1.2, CD19, and NK1.1. a) cDCs were gated as Thy1.2^-^ CD19^-^ NK1.1^-^ Siglec-H^-^ CD11c^+^ MHC-II^+^ cells. b) Macrophages were gated as Thy1.2^-^ CD19^-^ NK1.1^-^ CD11b^+^ CD64^+^ F4/80^+^ cells.

**
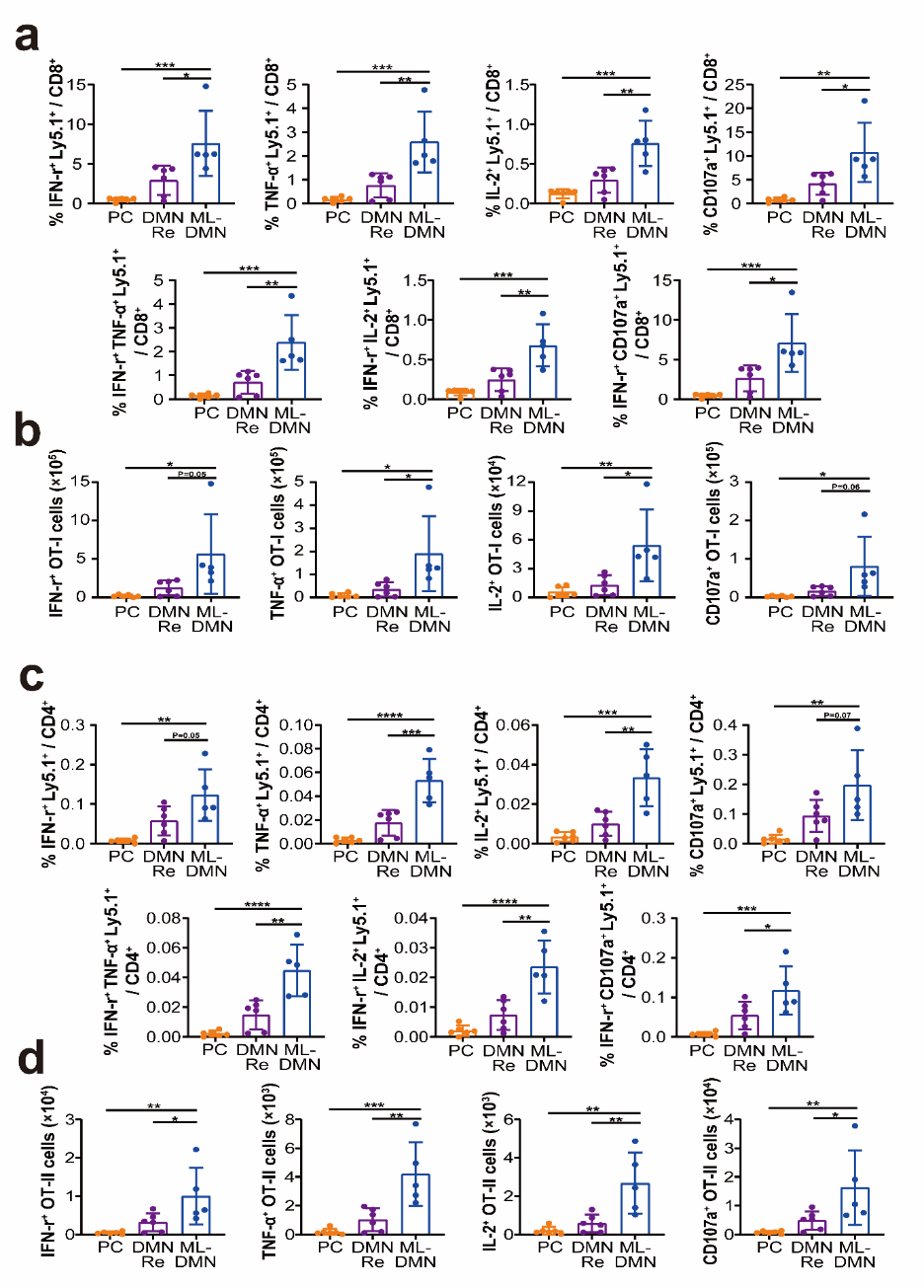
Figure S3.** Evaluation of effector function of antigen-specific T cells in mouse spleen by flow cytometry (n = 6–8 per group). As shown in Figure 5a, mice vaccinated on days 0 and 21 were sacrificed and the antigen-specific T cells present in the spleen were analyzed by flow cytometry on day 27. a) Graphs showing the frequencies of cytokine- and multi-cytokine-producing OT-I cells within CD8^+^ T cells. b) Graphs showing the absolute number of cytokine-producing OT-I cells. c) Graphs showing the frequencies of cytokine- and multi-cytokine-producing OT-II cells within CD4^+^ T cells. d) Graphs showing the absolute number of cytokine-producing OT-II cells. Data are representative of two independent experiments. Column graphs show mean ± SD. Statistical significance was analyzed by one-way ANOVA with Tukey’s multiple comparisons test. ns, not statistically significant; **p* < 0.05; ***p* < 0.01; ****p* < 0.001; *****p* < 0.0001.

**
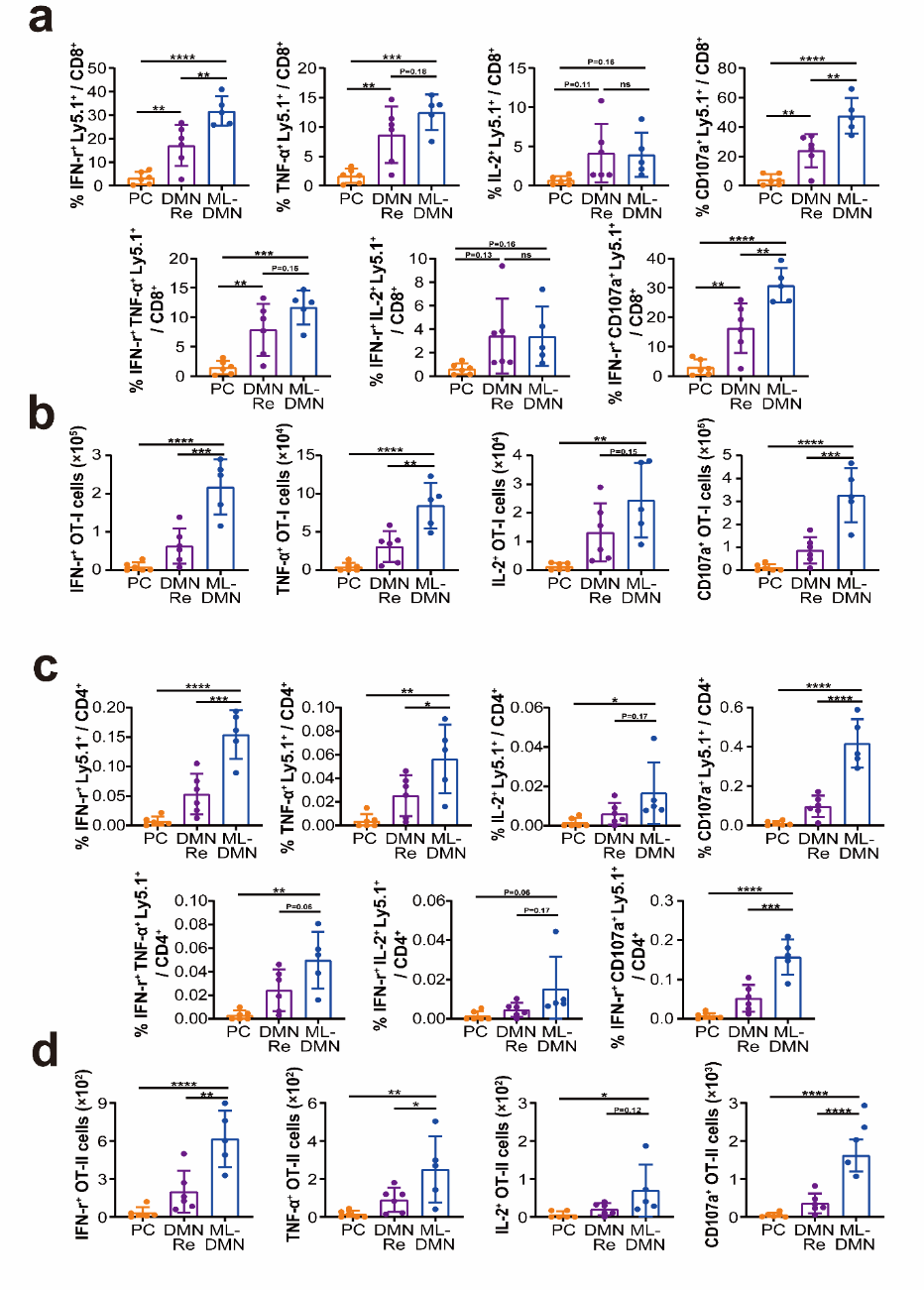
Figure S4.** Evaluation of effector function of antigen-specific T cells in mouse lung by flow cytometry (n = 6–8 per group). As shown in Figure 5a, mice vaccinated on days 0 and 21 were sacrificed and the antigen-specific T cells present in the lung were analyzed by flow cytometry on day 27. a) Graphs showing the frequencies of cytokine- and multi-cytokine-producing OT-I cells within CD8^+^ T cells. b) Graphs showing the absolute number of cytokine-producing OT-I cells. c) Graphs showing the frequencies of cytokine- and multi-cytokine-producing OT-II cells within CD4^+^ T cells. d) Graphs showing the absolute number of cytokine-producing OT-II cells. Data are representative of two independent experiments. Column graphs show mean ± SD. Statistical significance was analyzed by one-way ANOVA with Tukey’s multiple comparisons test. ns, not statistically significant; **p* < 0.05; ***p* < 0.01; ****p* < 0.001; *****p* < 0.0001.

**
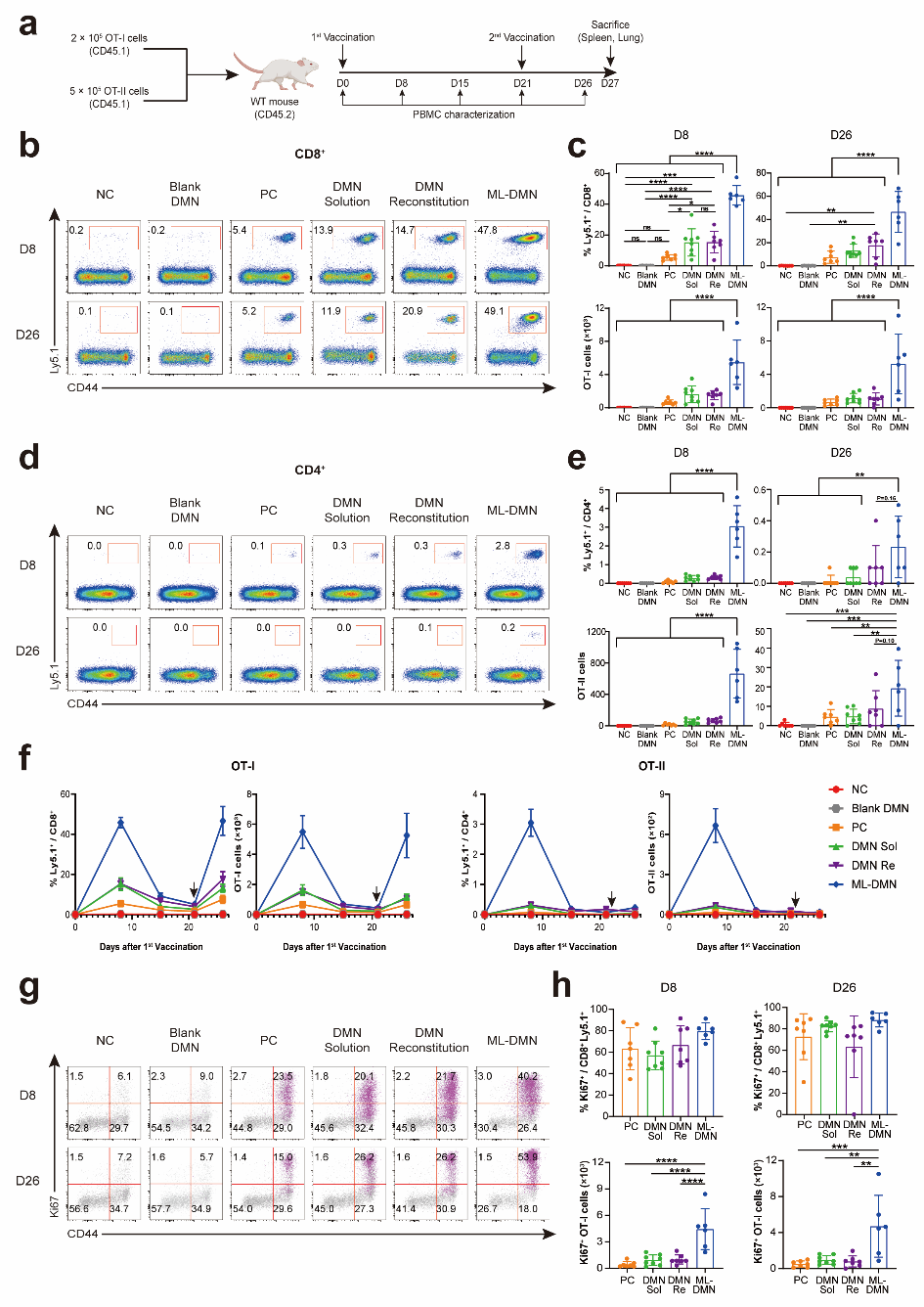
**

**Figure S5.** Experimental scheme and PBMC analysis at 3 µg OVA mRNA vaccination. a) *In vivo* experimental scheme for analysis of mRNA vaccine-induced immune responses. Naïve CD45.2^+^ mice (C57BL/6) were co-transferred with CD45.1^+^ OVA-specific OT-I (CD8^+^) and OT-II (CD4^+^) cells and then immunized with various forms of mRNA vaccines loaded with 3 µg OVA mRNA. Antigen-specific T cells in mouse PBMC and organs (spleen and lung) were analyzed by flow cytometry at indicated time points. b) Concatenated flow plots showing the frequencies of Ly5.1^+^ OT-I cells in PBMC on days 8 and 26, gated on CD8^+^ T cells. c) Graphs showing the frequencies (top) and cell number (bottom) of Ly5.1^+^ OT-I cells in PBMC. Data were normalized to 1 **×** 10^5^ PBMCs. d) Concatenated flow plots showing the frequencies of Ly5.1^+^ OT-II cells in PBMC on days 8 and 26, gated on CD4^+^ T cells. e) Graphs showing the frequencies (top) and cell number (bottom) of Ly5.1^+^ OT-II cells, normalized to 1 **×** 10^5^ PBMCs. f) Kinetics graphs showing dynamics in the frequencies and cell number of Ly5.1^+^ OT-I and OT-II cells after 1^st^ vaccination. The black arrows on the graphs indicate the boosting time point: Day 21. g-h) Ki67 expression of Ly5.1^+^ OT-I cells in PBMC was analyzed by flow cytometry. g) Concatenated flow plots showing the frequencies of Ki67^+^ CD8^+^ T cells in PBMC on days 8 and 26, gated on CD8^+^ T cells. The grey dots indicate the total CD8^+^ T cells and the purple dots indicate Ly5.1^+^ OT-I cells in PBMC. h) Graphs showing the frequencies (top) and number (bottom) of Ki67^+^ OT-I cells, normalized to 1 **×** 10^5^ PBMCs. Data are representative of two independent experiments. Column graphs show mean ± SD and kinetics graphs show mean ± SEM. Statistical significance was analyzed by one-way ANOVA with Tukey’s multiple comparisons test. ns, not statistically significant; **p* < 0.05; ***p* < 0.01; ****p* < 0.001; *****p* < 0.0001.

**
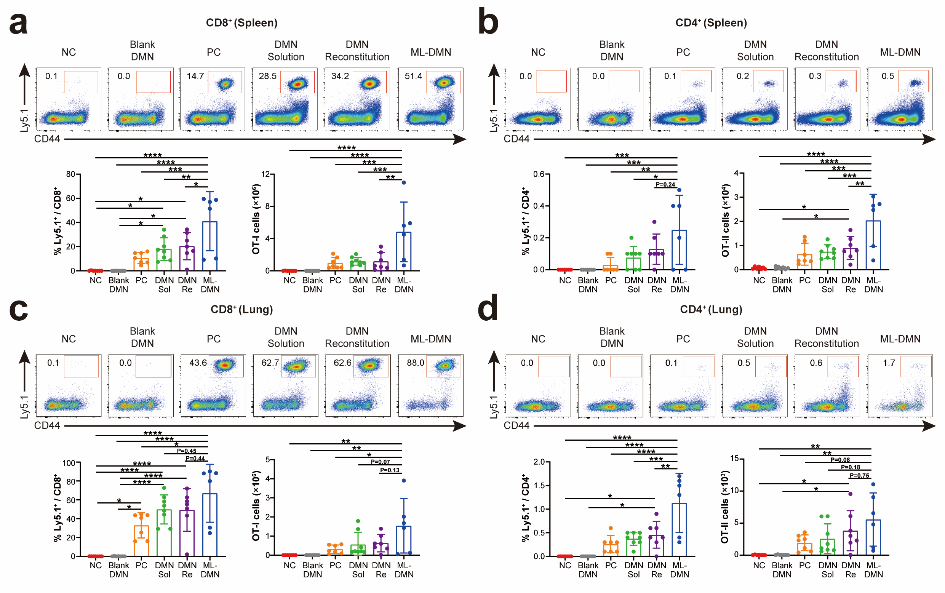
Figure S6.** Analysis of antigen-specific T cells in the spleen and lung of mice vaccinated with 3 µg OVA mRNA vaccines assessed by flow cytometry (n = 6–8 per group). As shown in Figure S5a, mice vaccinated on days 0 and 21 were sacrificed and the antigen-specific T cells present in the spleen and lung were analyzed by flow cytometry on day 27. a) Representative flow plots and graphs showing the frequencies and absolute number of Ly5.1^+^ OT-I cells in the spleen, gated on CD8^+^ T cells. b) Representative flow plots and graphs showing the frequencies and absolute number of Ly5.1^+^ OT-II cells in the spleen, gated on CD4^+^ T cells. c) Representative flow plots and graphs showing the frequencies and absolute number of Ly5.1^+^ OT-I cells in the lung, gated on CD8^+^ T cells. d) Representative flow plots and graphs showing the frequencies and absolute number of Ly5.1^+^ OT-II cells in the lung, gated on CD4^+^ T cells. Data are representative of two independent experiments. Column graphs show mean ± SD. Statistical significance was analyzed by one-way ANOVA with Tukey’s multiple comparisons test. ns, not statistically significant; **p* < 0.05; ***p* < 0.01; ****p* < 0.001; *****p* < 0.0001.

**
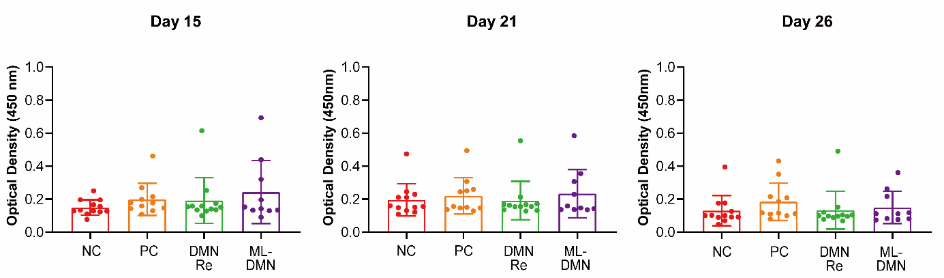
Figure S7.** Evaluation of anti-OVA IgG antibody levels in the serum of mice immunized with 1.5 µg OVA mRNA using ELISA (n=10–12 per group). As shown in Figure 5a, mice were vaccinated on days 0 and 21, and IgG antibody levels were analyzed at the specified time points. The ELISA plates coated with 10 μg/mL of full-length OVA protein were incubated with diluted serum (100×), and then the optical density was measured at 450 nm. Graphs show the relative amount of anti-OVA IgG antibody in mouse serum at the indicated time points. Data are representative of two independent experiments. Column graphs show mean ± SD. Statistical significance was analyzed by one-way ANOVA with Tukey’s multiple comparisons test. ns, not statistically significant.


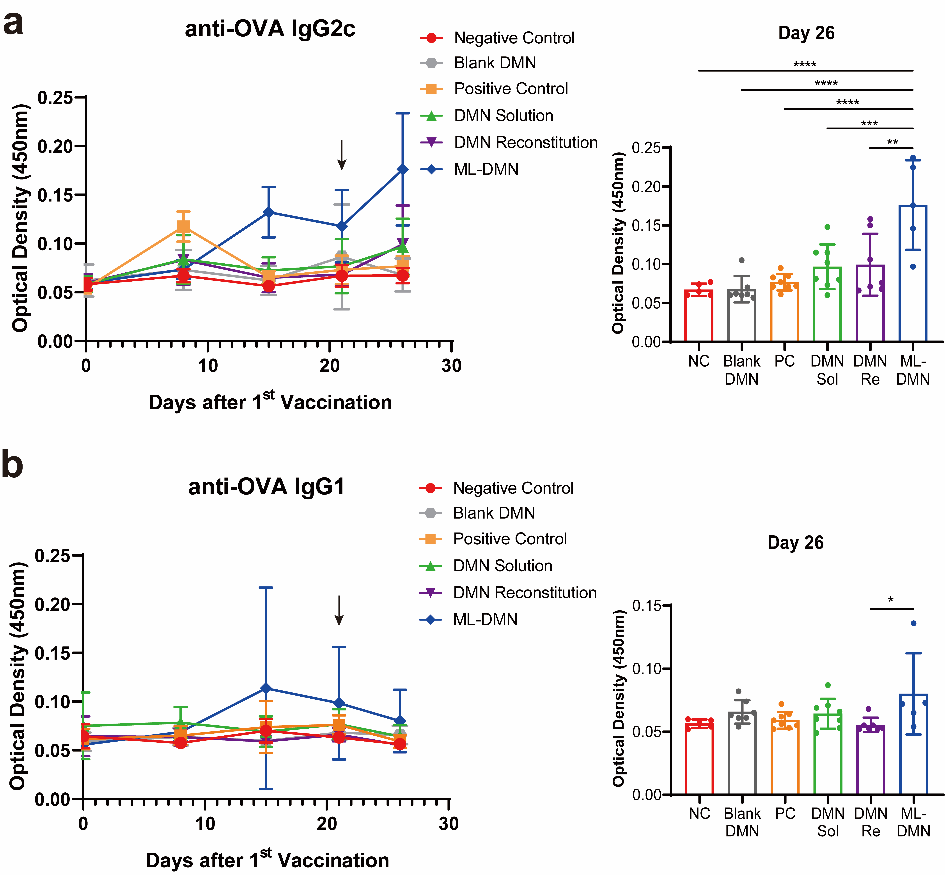


**Figure S8.** Serum levels of anti-OVA IgG2c and IgG1 antibodies were analyzed by ELISA in mice immunized with 3 µg OVA mRNA (n = 6–8 per group). ELISA plates coated with 10 µg mL⁻¹ full-length OVA protein were incubated with diluted serum (100×), and optical density was measured at 450 nm. a) Kinetic profile of anti-OVA IgG2c antibodies, representing Th1-associated humoral responses, following vaccination. The black arrow indicates the booster immunization on Day 21. The right panel shows the relative IgG2c antibody levels on Day 26. b) Kinetic profile of anti-OVA IgG1 antibodies, representing Th2-associated humoral responses, following vaccination. The black arrow indicates the booster immunization on Day 21. The right panel shows the relative IgG1 antibody levels on Day 26. Column graphs show mean ± SD, and kinetic graphs show mean ± SEM. Statistical significance was analyzed using one-way ANOVA with Tukey’s multiple comparisons test. ns, not statistically significant; **p* < 0.05; ***p* < 0.01; ****p* < 0.001; *****p* < 0.0001. Data are representative of two independent experiments.
